# N^6^‐Methyladenosine modification mediated by METTL3 promotes DNA‐PKcs expression to induce anlotinib resistance in osteosarcoma

**DOI:** 10.1002/ctm2.70228

**Published:** 2025-02-09

**Authors:** Yining Zhang, Guohong Shen, Dan Zhang, Tingting Meng, Zhaorui Lv, Lei Chen, Jianmin Li, Ka Li

**Affiliations:** ^1^ The First Clinical College of Cheeloo College of Medicine Shandong University Jinan Shandong China; ^2^ Department of Orthopaedics Qilu Hospital of Shandong University Jinan Shandong China; ^3^ Department of Pediatrics Central Hospital Affiliated to Shandong First Medical University Jinan Shandong China; ^4^ Department of Cardiovascular Medicine Jinan Central Hospital Jinan Shandong China; ^5^ Research Center of Translational Medicine Central Hospital Affiliated to Shandong First Medical University Jinan Shandong China; ^6^ Department of Orthopedics Affiliated Hospital of Shandong University of Traditional Chinese Medicine Jinan China

**Keywords:** anlotinib, DNA‐PKcs, m^6^A, METTL3, osteosarcoma

## Abstract

**Background:**

Acquired anlotinib resistance is still a key challenge in osteosarcoma treatment. Unravelling the mechanisms underlying anlotinib resistance is the key to optimising its efficacy for treating osteosarcoma. Previous studies have explored the pivotal function of the DNA‐dependent protein kinase catalytic subunit (DNA‐PKcs) with regard to osteosarcoma chemoresistance.

**Methods:**

We used bioinformatics analysis to predict DNA‐PKcs and Beclin‐1 interactions, confirmed through immunofluorescence (IF) and co‐immunoprecipitation (co‐IP). Dual‐luciferase analyses and Methylated RNA immunoprecipitation (MeRIP) were implemented to detect the detected m^6^A modifications. RNA fluorescence in situ hybridisation (FISH)—IF co‐localisation and RNA immunoprecipitation (RIP) were conducted to explore the interplay between PRKDC mRNA and the indicated proteins.

**Results:**

Anlotinib‐treated osteosarcoma cells exhibited increased DNA‐PKcs levels, and silencing DNA‐PKcs augmented osteosarcoma sensitivity to anlotinib. DNA‐PKcs affects anlotinib‐induced autophagy by interacting with Beclin‐1 and regulating its ubiquitination. Notably, PRKDC mRNA, encoding DNA‐PKcs, underwent N^6^‐Methyladenosine (m^6^A) modification. Methyltransferase‐like 3 (METTL3) positively regulated DNA‐PKcs expression. Functionally, METTL3 enhances anlotinib resistance in osteosarcoma, which is reversed by PRKDC knockdown. Mechanistically, METTL3 binds to PRKDC mRNA and facilitates m^6^A methylation. Additionally, m^6^A methylated PRKDC mRNA is identified via YTH N^6^‐methyladenosine RNA‐binding protein 1 (YTHDF1), augmenting its expression.

**Conclusion:**

These findings revealed that DNA‐PKcs promotes anlotinib resistance by regulating protective autophagy, while METTL3 induces PRKDC m^6^A modification, enhancing its expression. Thus, targeting METTL3/PRKDC may be a novel strategy for improving therapeutic efficacy in human osteosarcoma.

**Key points:**

- DNA‐PKcs knockdown heightens osteosarcoma sensitivity to anlotinib.
- DNA‐PKcs modulates anlotinib‐induced protective autophagy through interacts with Beclin‐1 and regulates its ubiquitination.
- m^6^A modification of OLE_LINK82PRKDC mRNA induced by METTL3 contributes to anlotinib resistance in osteosarcoma.
- m^6^A methylation of PRKDC mRNA recognised by YTHDF1 amplifies the expression of DNA‐PKcs.

## Graphical abstract

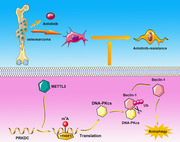

## 1 INTRODUCTION

As the most widespread bone cancer among young individuals, osteosarcoma is derived from primitive mesenchymal stem cells.^1^ It primarily affects children and adolescents and shows a high propensity for local tissue invasion and early metastasis, with a recurrence rate of approximately 30–50%.^1^, ^2^ Although aggressive, multi‐drug chemotherapy regimens may yield improved treatment results, they also significantly increase chemotherapy toxicity levels and create challenges in addressing drug resistance.^3^, ^4^ Targeted drug therapy is a crucial treatment strategy for patients with conventional chemotherapy limitations or advanced‐stage osteosarcoma.

Anlotinib, a recently developed oral multitarget tyrosine kinase suppressor, targets vascular endothelial growth factor receptors (VEGFR) and fibroblast growth factor receptors (FGFR), among others, thereby inhibiting angiogenesis and tumour progression.^5^, ^6^ It is approved as a third‐line option for managing non‐small cell lung cancer (NSCLC). It is highly recommended to treat advanced solid tumours, such as medullary thyroid cancer, colon adenocarcinoma, renal clear cell cancer, and soft tissue sarcomas.^6^, ^7^, ^8^, ^9^ Research indicates that Anlotinib markedly reduces osteosarcoma growth and metastasis by suppressing MET and VEGFR2 phosphorylation.^5^ A multi‐institutional retrospective study revealed a potent inhibitory action of anlotinib against metastatic or unresectable osteosarcoma.^10^ Furthermore, anlotinib has also been utilised to integrate chemotherapy and radiotherapy to treat relapse osteosarcoma.^11^ Liu et al. also demonstrated its potential to reverse multi‐drug resistance in osteosarcoma.^12^ Thus, anlotinib is a promising therapeutic option for advanced or chemoresistant osteosarcomas.

Despite advancements in tumour therapy, acquired drug resistance remains a critical issue that frequently leads to treatment failure. Several studies have confirmed that the TFAP2A gene and exosomal miR‐136‐5p of non‐small cell lung cancer (NSCLC) are resistant to anlotinib, whereas anlotinib resistance in gastric cancer is promoted by cancer‐associated fibroblasts (CAFs) by reducing anlotinib‐induced ROS.^13^, ^14^, ^15^ Liang et al. demonstrated that anlotinib induces protective autophagy in NSCLC and that blocking autophagy increases the efficacy of anlotinib.^6^ Additionally, Tocilizumab (an anti‐IL‐6R monoclonal antibody) and PI3K inhibitors enhance the sensitivity of anlotinib‐resistant osteosarcoma cells.^16^, ^17^ Given the limited research on the mechanism of anlotinib resistance in osteosarcoma, further investigation is essential to provide theoretical insights and improve therapeutic efficacy.

N^6^‐methyladenosine (m^6^A), the most frequent mRNA modification, modulates gene expression by influencing RNA processing, translation, and degradation.^18^, ^19^, ^20^ This is facilitated through the interplay between ‘writers’, ‘erasers’, and ‘readers’.^21^ Multiple studies have revealed the pivotal role of methyltransferase‐like 3 (METTL3), an RNA methyltransferase with respect to the progression of tumours, such as osteosarcoma, bladder cancer, and glioblastoma.^22^, ^23^, ^24^ Studies have demonstrated that METTL3 promotes the initiation and metastasis of oral squamous cell carcinoma through m^6^A modification.^25^, ^26^ In particular, METTL3 controls ATAD2 expression through m^6^A modification in osteosarcoma, thus boosting tumour growth, migration, and invasion.^22^ However, the relationship between METTL3 expression and anlotinib resistance in osteosarcoma remains unclear.

Prior research has emphasised the key function of DNA‐PKcs in osteosarcoma recurrence, metastasis, and chemoresistance.^27^, ^28^ Additionally, eicosapentaenoic acid (EPA) inhibits the DNA‐PKcs/AKT and DNA‐PKcs/PD‐L1 pathways, thereby enhancing the therapeutic impact of DDP in osteosarcoma.^29^ In this investigation, a novel discovery was made regarding the elevation of DNA‐PKcs expression after anlotinib treatment. Consequently, we aimed to provide strong evidence to determine whether DNA‐PKcs affect the sensitivity of osteosarcoma to anlotinib.

This investigation was focused on unravelling the molecular mechanisms bringing about anlotinib resistance in osteosarcoma. We aimed to determine whether DNA‐PKcs modulate osteosarcoma sensitivity to anlotinib and to elucidate the underlying mechanisms. We also investigated the role of METTL3‐induced m^6^A modifications in DNA‐PKcs expression. Our research seeks to present a novel approach for future clinical studies on acquired drug resistance in osteosarcoma.

## 2 MATERIALS AND METHODS

### 2.1 Cell lines and culture

MG63 and U‐2 OS cells were supplied by the American Type Culture Collection (ATCC; Manassas, VA, USA). The cells were seeded within Dulbecco's minimal essential medium (DMEM, Gibco, USA) supplemented with 10% fetal bovine serum (FBS, Gibco, USA) and 1% antibiotics (Solarbio, China), maintained under a 95% moist environment at 37°C with 5% CO_2_.

### 2.2 Drug preparations and reagents

Anlotinib (Selleck Chemicals, Houston, TX, USA) was dissolved in physiological saline and diluted in the culture medium to achieve the desired concentrations.

### 2.3 Plasmids and small interfering RNA (siRNA) transfections

Keyybio (Shandong, China) synthesised the METTL3 and negative control plasmids, whereas RiboBio (Guangzhou, China) produced siRNAs targeting PRKDC, METTL3, YTHDF1, and YTHDF2. The cell were fostered within 6‐ or 24‐well plates till reaching 50–70% confluence before transfection with plasmids and siRNAs through Opti‐MEM (Gibco) and Lipofectamine 2000 (Invitrogen, ThermoFisher, China) according to the manufacturer's instructions. Subsequent experiments were conducted 48–72 h later. The plasmid and siRNA sequences are listed in Tables S1 and S2.

### 2.4 RNA isolation and quantitative real‐time PCR (qRT‐PCR) analysis

TRIzol lysis buffer (Toyobo, Japan) was utilised to obtain the total RNAs, followed by reverse transcription of the isolated RNA to cDNA through the Evo M‐MLV RT Mix Kit with gDNA Clean (Accurate Biology, Hunan, China), complying with the manufacturer's specifications. qRT‐PCR was implemented through the SYBR®Green PremixPro TaqHS qPCR Kit (Accurate Biology) analysed on the QuantStudio™ Real‐Time PCR System (ThermoFisher, China). GAPDH served as internal control, with the comparative expression of target genes was determined using the 2^−ΔΔCT^ method. Table 1 displays the primer pairs for qRT‐PCR.

**TABLE 1:** The primers sequences of genes used in qRT‐PCR.

Gene	Primer sequence, 5′–3′	
	Forward	Reverse
PRKDC	GCCCACCCTCTTGTACCTTC	GCTCCTACAGTTCTCTCGCC
Beclin‐1	AACCGCAAGATAGTGGCAGA	CTCTCTGATACTGAGCTTCCTCC
METTL3	TGATGCTGATCGACCCTGTC	CTTGGCGTGTGGTCTTTGC
METTL14	GGCAGAAGTTACGGCGACAG	ATTTAACACGGCACCAATGCT
YTHDF1	CTTCAGCGTCAATGGGAGTG	CCGGAGCTGGTTATTGGGTA
YTHDF2	ACAAGAGACTGGATGCTGCT	TTGGCTATTGGGAACGTCCT
GAPDH	GCACCGTCAAGGCTGAGAAC	TGGTGAAGACGCCAGTGGA

### 2.5 Protein extraction and Western blotting analysis

Tumour tissues or cells were dissolved in RIPA buffer (Beyotime, China) consisting of protease and phosphatase suppressors (Solarbio) for half an hour. The protein concentrations were quantified using the BCA protein assay kit (Beyotime Biotechnology). The same quantity of proteins were segregated by sodium dodecyl sulphate‐polyacrylamide gel electrophoresis (SDS‐PAGE, Epizyme Biotech, Shanghai, China) and delivered to polyvinylidene fluoride (PVDF) membranes (Millipore, USA). The membranes experienced a blocking step with 5% nonfat dry milk for 1 h at room temperature (RT) before incubation at 4°C with primary antibodies all night. Following washed through PBST (phosphate‐buffered saline containing 0.2% Tween‐20), the secondary antibodies were utilised for the membranes and left to incubate for 1 h at RT. Immunoreactive bands were checked through an enhanced chemiluminescence substrate (EMD Millipore, Billerica, USA), and the images were captured using one chemiluminescence apparatus (Tanon 5200Multi, China).

#### 2.5.1 Antibodies used were listed below

Primary antibodies were DNA‐PKcs (1:500, Abcam, UK, ab70250), Bcl‐2 (1:1000, Santa Cruz, USA, sc‐56018), Bax (1:1000, Proteintech, Wuhan, China, 50599‐2‐Ig), cleaved‐caspase3 (1:200, Cell Signaling Technology, USA, 9661), caspase3 (1:1000, Cell Signaling Technology, 9662), Beclin‐1 (1:1000, Proteintech, 66665‐1‐Ig), p62 (1:1000, Proteintech, 18420‐1‐AP), LC3 (1:1000, Proteintech, 14600‐1‐AP), Ubiquitin (1:1000, Proteintech, 10201‐2‐AP), METTL3 (1:1000, Proteintech, 15073‐1‐AP), YTHDF1 (1:1000, Proteintech, 17479‐1‐AP), YTHDF2 (1:1000, Proteintech, 26771‐1‐AP) and β‐actin (1:5000, ZSGB‐Bio, Beijing, China, TA‐09). Secondary antibodies used were goat anti‐mouse IgG (H + L) (1:5000, ZSGB‐Bio) and goat anti‐rabbit IgG (H + L) (1:5000, ZSGB‐Bio).

### 2.6 Cell viability assay and colony formation assay

To conduct the test of cell activity, cells were fostered in 96‐well plates and underwent specific treatments for 24, 48, 72, or 96 h at 37°C. Subsequently, cell activity was assessed through Cell Counting Kit‐8 (CCK‐8, Dojindo, Japan) complying with the manufacturer's guidelines.

To experiment colony formation, the cells were put within 6‐well plates and brooded under specified conditions for 10–14 days at 37°C. Afterwards, the cells were immobilised in 4% paraformaldehyde for half an hour, coated with 0.1% crystal violet for half an hour at RT, and subsequently graphed to capture the colony formation.

### 2.7 5‐Ethynyl‐2′‐Deoxyuridine (EdU) assay

Following plating within 24‐well plates, the cells were transfected with si‐PRKDC and treated with anlotinib for 24 h. Following the manufacturer's guidelines, the Cell‐Light™ EdU Apollo 567 In Vitro Kit (RiboBio, Guangzhou, China) was used for the EdU assay. Observations were performed using upright fluorescence microscopy (Olympus, Tokyo, Japan).

### 2.8 Transwell assays

After the specified treatment, the cells were dissolved, rinsed with phosphate‐buffered saline (PBS), suspended at a density of 5 × 10^4^ cells/200 µL again, and put in the upper chambers of 24‐well plates (Corning, NY, USA) containing serum‐free DMEM. Matrigel (Corning) was applied to stain the chambers for invasion assays following the manufacturer's guidelines. Subsequently, DMEM integrated into 10% FBS was placed into the lower compartment. Following 1 day, the upper compartment contents were eliminated through cotton swabs, following that, the cells located underneath the upper compartments were treated through 4% paraformaldehyde for 30 min and rinsed using PBS. Afterwards, 0.1% crystal violet solution was applied to cells for 15 min at room temperature, before image acquisition through a photon microscope (Olympus, Japan).

### 2.9 Apoptosis analysis

Cells were cultured within 24‐well plates, transfected using si‐PRKDC, and treated with anlotinib for 24 h. Subsequently, MG63 and U‐2 OS cells were immobilised using 4% paraformaldehyde, rinsed using PBS, and permeabilised using 0.1% Triton X‐100 (Solarbio). Following the manufacturer's guidelines, an apoptosis assay kit based on the TUNEL assay was applied to recognise apoptotic cells. Observations were performed using an upright fluorescence microscope (Olympus, Japan).

Flow cytometry was applied to rate the proportion of apoptosis cells through an Annexin V‐FITC/PI apoptosis detection kit (YEASEN, China) as per the manufacturer's specifications. Data analysis was performed using FlowJo software.

### 2.10 Lentivirus infection

PRKDC short hairpin RNA (shRNA), control shRNA, METTL3, and control lentiviruses were synthesised by GeneChem Co. (Shanghai, China). MG63 cells were infected with the suitable lentiviruses complying with to the manufacturer's protocol. Following 2 or 3 days of incubation, cells with stable expression were selected using puromycin or neomycin.

### 2.11 Tumour xenografts and animal experiments

BALB/c nude mice (male, 5 weeks old) were supplied by Vital River Laboratory Animal Technology Co., Ltd. (Beijing, China) and maintained under specific pathogen‐free (SPF) conditions with a light‐dark cycle for 12 h. Following 1‐week acclimatisation, a suspension containing 5 × 10^6^ stably expressing MG63 cells was injected subcutaneously into the right axilla of each mouse. If tumours was palpable, the mice were allocated stochastically to two groups, one as the control (CTR) group while the other received anlotinib treatment (3 mg/kg/day for 2 weeks via intragastric administration). The dimensions of the xenograft tumours were gauged every third day. The tumour volume was figured out through the formula: volume (mm^3^) = length × width^2^ × 0.5. After 2 weeks of treatment, following the experimental procedures, the mice were euthanised. The tumour tissues were eliminated, photographed, and then weighed. The xenograft tumours were immobilised within in 4% paraformaldehyde for subsequent haematoxylin and eosin (H&E) and immunohistochemical (IHC) staining, whereas other portions were frozen for protein extraction.

All experimental procedures complied with animal care standards of the Chinese National Institute of Health and received approvals from the Research Ethics Committee of the Shandong University Qilu Hospital.

### 2.12 Haematoxylin‐eosin staining (H&E) and immunohistochemistry (IHC)

Xenograft tumour tissues harvested from mice were immobilised within 4% paraformaldehyde, placed into paraffin, followed by cutting into 4 µm slices. Subsequently, the slices were deparaffinised with an environmental‐friendly transparent dewaxing liquid (Solarbio) and hydrated using an array of ethanol solutions (100 %, 95 %, 90 %, 80 %, and 70 %). Slices were processed for haematoxylin and eosin staining (Servicebio, Wuhan, China). For IHC staining, the tissue samples were sensitive to retrieving antigen using a Sodium Citrate Antigen Retrieval Solution (Solarbio) or EDTA antigen retrieval solution (pH 9.0; ZSGB‐Bio). IHC staining was implemented following the manufacturer's instructions (PV‐8000, ZSGB‐Bio). Images were captured through one upright microscope (Olympus, Japan).

#### 2.12.1 Antibodies were listed below

DNA‐PKcs (1:200, Abcam), Ki67 (1:100, Proteintech), cleaved‐caspase3 (1:100, Cell Signaling Technology), Beclin‐1 (1:200, Proteintech), p62 (1:200, Proteintech) and METTL3 (1:200, Proteintech).

### 2.13 RFP‐GFP‐LC3 lentivirus infection and autophagy analyses

RFP‐GFP IRES‐Puromycin Human MAP1LC3B lentivirus was synthesised by Genechem Co. (Shanghai, China). MG63 and U‐2 OS cells were stably infected according to the protocol for lentivirus infection. Following the stable infection, these cells were cultured within 24‐well plates and underwent specific treatments. After that, the cells underwent a wash with PBS and were subsequently fixed through 4% paraformaldehyde for half an hour at RT. The slides were sealed with an Antifade Mounting Medium after coating the nuclei using DAPI (Beyotime Biotechnology). An upright fluorescence microscope (Olympus, Japan) was employed to capture images.

### 2.14 Transmission electron microscopy

After collection, the cells underwent centrifugation at 300 × *g* for 5 min, then fixed with 2.5% glutaraldehyde (Solarbio) for half an hour at RT. The electron microscopy facility at Jinan Central Hospital performed post‐fixing, embedding, sectioning, and mounting. The samples were then examined via transmission electron microscopy to observe autophagosomes.

### 2.15 Human osteosarcoma tissue collection

All human osteosarcoma tissues were obtained from postoperative patients at Qilu Hospital of Shandong University. None of the patients were treated for any anticancer. Informed consent was derived from the total participators, with the protocols receiving approvals from the Research Ethics Committee of Shandong University Qilu Hospital.

### 2.16 Immunofluorescence (IF)

After incubating within 24‐well plates for 1 day, cells were sensitive to fixation through 4% paraformaldehyde for 15 min, permeabilised with 0.5% Triton X‐100 (Solarbio) for 10 min, blocked with 10% healthy goat serum (Solarbio) for half an hour, and then brooded at 4°C using rabbit anti‐DNA‐PKcs (1:200, Abcam) and mouse anti‐Beclin‐1 (1:200, Proteintech) antibodies all night. Afterwards, the cells were rinsed and brooded with Alexa Fluor® 488 goat anti‐rabbit IgG (1:200, Abcam) and Alexa Fluor® 594 goat anti‐mouse IgG (1:200, Abcam) antibodies for 1 h at RT. Antifade Mounting Medium with DAPI (Beyotime) was applied to stain the nuclei and seal the slides. Sections of human osteosarcoma tissue were deparaffinised, hydrated, and sensitive to retrieving antigens by heating. After blocking with 10% normal goat serum, antibody incubation was performed, as described in the cell protocol. Images were captured through one upright fluorescence microscope (Olympus, Japan).

### 2.17 Co‐Immunoprecipitation (co‐IP) and ubiquitination analysis

Cells and human osteosarcoma tissue were collected to assess the interactions between DNA‐PKcs and Beclin‐1. Co‐IP was performed using a Co‐IP Kit (Bersin Bio, China) following the manufacturer's guidelines. Antibodies against DNA‐PKcs (5 µg, Abcam) and Beclin‐1 (5 µg, Proteintech) were used. To examine ubiquitination, cells or xenograft tumour tissues were treated as specified and then lysed, followed by the incubation of lysates with an anti‐Beclin‐1 antibody (5 µg, Proteintech). After the completion of the co‐IP process, western blot analysis was performed on the immunoprecipitates.

### 2.18 RNA fluorescence in situ hybridisation (FISH)—IF

To detect PRKDC mRNA co‐localisation with target proteins, MG63 and U‐2 OS cells were plated in 24‐well plates, while human osteosarcoma tissues underwent fixation, embedding, and sectioning into 4 µm slices. Cy‐5 conjugated PRKDC mRNA probes were devised and compounded by Bersin Bio (Guangzhou, China). Subsequent steps included FISH and IF using an Immunofluorescence‐Fluorescent in situ hybridisation (IF‐FISH) Kit (Bersin Bio), following the manufacturer's guidance.

#### 2.18.1 Antibodies applied to immunofluorescence were listed below

Rabbit anti‐N^6^‐methyladenosine antibody (1:200, ABclonal, Wuhan, China, A17924), rabbit anti‐METTL3 antibody (1:200, Proteintech), rabbit anti‐YTHDF1 antibody (1:200, Proteintech) and Alexa Fluor®488 goat anti‐rabbit IgG (1:200, Abcam).

### 2.19 Methylated RNA immunoprecipitation (MeRIP)‐qPCR

Cells and human osteosarcoma tissues were collected and washed twice with PBS. TRIzol lysis buffer was used for obtaining the total RNAs. After fragmenting total RNA, assessing the m^6^A modification of PRKDC mRNA was implemented through the methylated RNA immunoprecipitation (MeRIP) Kit (BersinBio, China) and N^6^‐methyladenosine antibody (5 µg, ABclonal) as per the manufacture protocols. The RNA was first grouped and incubated with an N^6^‐methyladenosine antibody for 4 h. After incubation, pre‐prepared protein A/G magnetic beads were integrated. The mixture was brooded for extra 1 h to allow antibody‐sample hybridisation. After extensive washing, elution was performed using the Elution Buffer provided with the kit. The RNA eluted was obtained through phenol‐chloroform‐isoamyl alcohol (25:24:1) (Solarbio). RNA enrichment was quantified by qRT‐PCR. Table 2 displays the primer pairs for MeRIP‐qPCR.

**TABLE 2:** The primers sequences of genes used in MeRIP‐qPCR.

Gene	Primer sequence, 5′–3′	
	Forward	Reverse
PRKDC P1	TGGTGGCGAAAAATGCAGAA	ACGGTCCTGCAAAAAGTCCA
PRKDC P2	ACAGCAAATGCACCGTTGTG	GCACCAGGACTCTCATCAGG
PRKDC P3	AAAGAAGTGTATGCCGCTGC	TCGCAACCAGTTCACACAGA
PRKDC P4	AAAGACTCAAAGCCCCCTGG	CTGGCAATGGCTTTCCCCTA

### 2.20 RNA immunoprecipitation (RIP) assay

Cells and human osteosarcoma tissues were collected, rinsed using PBS, and dissolved with one lysis buffer consisting of a protease suppressor cocktail and an RNase inhibitor. Subsequently, an RNA immunoprecipitation kit (Geneseed Biotech, Guangzhou, Guangdong, China) was used for RIP assay. The required antibodies were brooded using the protein A+G beads for 2 h, followed by two washes. The resulting antibody‐bead complexes were combined with cell or tissue lysates and incubated at 4°C all night. After rinsed extensively, proteins and RNA were eluted through the elution buffer provided with the kit. Western blot analysis was conducted for protein enrichment, and RNA enrichment was quantified using qRT‐PCR.

#### 2.20.1 Antibodies used were as follows

METTL3 (5µg, Proteintech) and YTHDF1 (5µg, Proteintech). qRT‐PCR was used to analyse RNA enrichment.

### 2.21 Dual‐luciferase reporter analysis

PmirGLO Dual‐Luciferase Reporter plasmids, which included the wild‐type or mutated sequences of the four predicted areas of PRKDC mRNA, were constructed using Keyybio (Shandong, China). MG63 cell cultures were initiated in 24‐well plates and co‐transfected with the luciferase vector along with METTL3 plasmids, METTL3 siRNA, or a negative control. Following a 48‐h incubation period, the firefly and Renilla luciferase activities were quantified through the Promega Dual‐Luciferase System (Promega, USA) according to the manufacturer's guidelines. Data were collected using a Centro XS3 LB 960 (Berthold, Germany) and MikroWin software.

### 2.22 Statistical analysis

GraphPad Prism v8.0 (La Jolla, USA) and SPSS v22.0 (IBM, USA) were applied to statistical analyses. Analysts conducting the statistical assessments were blinded to the treatment groups. Each trial was replicated at least thrice. Data were presented as means ± standard deviation (SD), with contrasts between two groups construed through the Student's *t*‐test, whereas one‐way analysis of variance (ANOVA) with Bonferroni correction was applied to identify significant disparities between groups. *p* < .05 held statistical significance.

## 3 RESULTS

### 3.1 DNA‐PKcs expression was relevant to the sensitivity of osteosarcoma to anlotinib

To examine the potential engagement of DNA‐PKcs in the response of osteosarcoma cells to anlotinib, MG63 and U‐2 OS cell lines were treated using anlotinib. The PRKDC mRNA and DNA‐PKcs protein levels were rated through qRT‐PCR and western blotting, respectively. As shown in Figure S1A, a notable rise in PRKDC mRNA levels was observed in the anlotinib‐treated group. Following 24–96 h of anlotinib treatment, a slight reduction occurred in the level of DNA‐PKcs, which was then followed by a significant increase after 72 and 96 h of treatment (Figure S1B).

**FIGURE 1:**
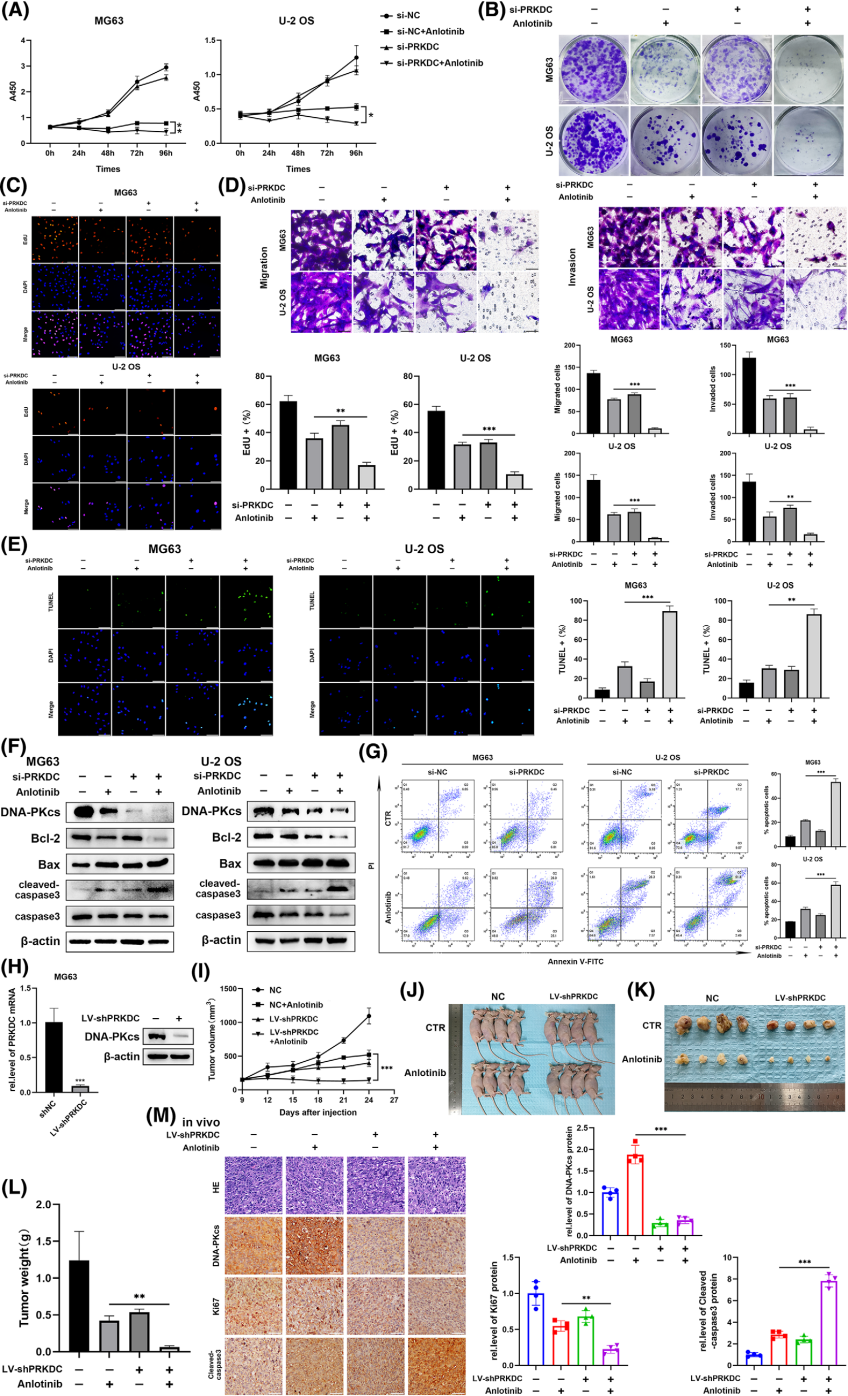
DNA‐PKcs correlate with osteosarcoma cell sensitivity to anlotinib both in vitro and in vivo. MG63 and U‐2 OS cells were transfected with PRKDC siRNA or control siRNA before being treated with anlotinib. (A) The CCK8 assay assessed the viability of MG63 and U‐2 OS cells after the indicated treatments. (B) Plate colony formation assay of MG63 and U‐2 OS cells. (C) EdU staining evaluated the proliferation of MG63 and U‐2 OS cells following the treatment with anlotinib for 24 h, scale bar = 100µm. (D) Transwell assay, without or with Matrigel, determined the migration and invasion capabilities of the cells after anlotinib treatment for 24 h, scale bar = 50µm. (E) TUNEL staining compared apoptosis induction between si‐NC and si‐PRKDC transfected cells after anlotinib treatment for 24 h, Scale bar = 100µm. (F) Western blotting assessed protein levels of DNA‐PKcs, Bcl‐2, Bax, cleaved‐caspase3, and caspase3 in MG63 and U‐2 OS cells transfected with si‐PRKDC and treated with anlotinib for 24 h. (G) Flow cytometry quantified the percentage of apoptotic cells after the specified intervention. (H) qRT‐PCR and western blotting analysed PRKDC mRNA and DNA‐PKcs protein expression in PRKDC shRNA lentivirus or control shRNA lentivirus‐stably infected MG63 cells. Stable MG63 cells were injected into nude mice and treated with anlotinib. (I) Tumour volume was measured every three days. (J) Mice were sacrificed, and representative images of the gross MG63 tumours were captured at the end of the experiment. (K) Representative photographs of xenograft tumours. (L) MG63 xenograft tumour weights were measured. (M) Representative images of H&E and IHC staining for DNA‐PKcs, Ki67, and cleaved‐caspase3 in the tumours, scale bar = 50µm. Data are presented as mean ± SD, *n* = 3. *p < .05; **p < .01; ***p < .001 (Student's *t*‐test for two‐group comparisons, one‐way ANOVA with Bonferroni post‐tests for multiple group comparisons).

To evaluate the influence of DNA‐PKcs expression on the response elicited by anlotinib in osteosarcoma cells, MG63 and U‐2 OS cells were transfected through PRKDC siRNA, followed by treatment with anlotinib. Enhanced anlotinib‐induced inhibition of cell viability was notably observed in the DNA‐PKcs siRNA group, as indicated by the CCK8 assay (Figure 1A). Subsequently, the colony formation assays indicated a remarkable increase in the efficacy of the anlotinib‐induced reduction in cell proliferation due to DNA‐PKcs knockdown, corresponding to findings from the EdU staining assays (Figure 1B and C). Similarly, the si‐PRKDC+ anlotinib group exhibited a distinct decrease in the metastasis and invasion of MG63 and U‐2 OS cells relative to the anlotinib group, as illustrated in Figure 1D. TUNEL staining revealed an essential rise in anlotinib‐induced apoptosis among osteosarcoma cells following DNA‐PKcs knockdown (Figure 1E). Consistent with these findings, the levels of the anti‐apoptotic protein Bcl‐2 were notably reduced, whereas there was a marked elevation in the level of the pro‐apoptotic proteins Bax and cleaved‐caspase3 in MG63 and U‐2 OS cells treated with si‐PRKDC+ anlotinib, as opposed to treatment with anlotinib alone (Figure 1F). Furthermore, there was an increase in the percentage of apoptotic osteosarcoma cells in the si‐PRKDC+ anlotinib group relative to that in the group receiving anlotinib alone, as detected via flow cytometry (Figure 1G).

To examine the impact of DNA‐PKcs on the reaction of osteosarcoma cells to anlotinib in vivo, xenograft tumour models were formulated among nude mice through MG63 cells steadily transfected using either PRKDC shRNA or a control shRNA lentivirus. The efficiency of shRNA lentivirus transfection was confirmed, as shown in Figures 1H and s1c. As shown in Figure S1D–F, consistent with the effects observed following si‐PRKDC treatment, PRKDC shRNA significantly potentiated the reaction of osteosarcoma cells to anlotinib. Following confirmation of shRNA lentivirus transfection efficiency, the mice were segregated into control (CTR) and anlotinib‐treated groups. Our results revealed that DNA‐PKcs knockdown enhanced the antitumour effects of anlotinib (Figure 1I–L). Notably, IHC staining of tumour xenograft tissues indicated that the shPRKDC+anlotinib combination led to a notable reduction in Ki67 expression and an elevation in cleaved‐caspase3 levels when compared to the anlotinib group transfected with the control shRNA lentivirus (Figure 1M).

These results confirmed that the downregulation of DNA‐PKcs markedly enhanced anlotinib‐induced responses in osteosarcoma cells both in vitro and in vivo.

### 3.2 DNA‐PKcs was associated with autophagy response through interact with Beclin‐1

**FIGURE 2:**
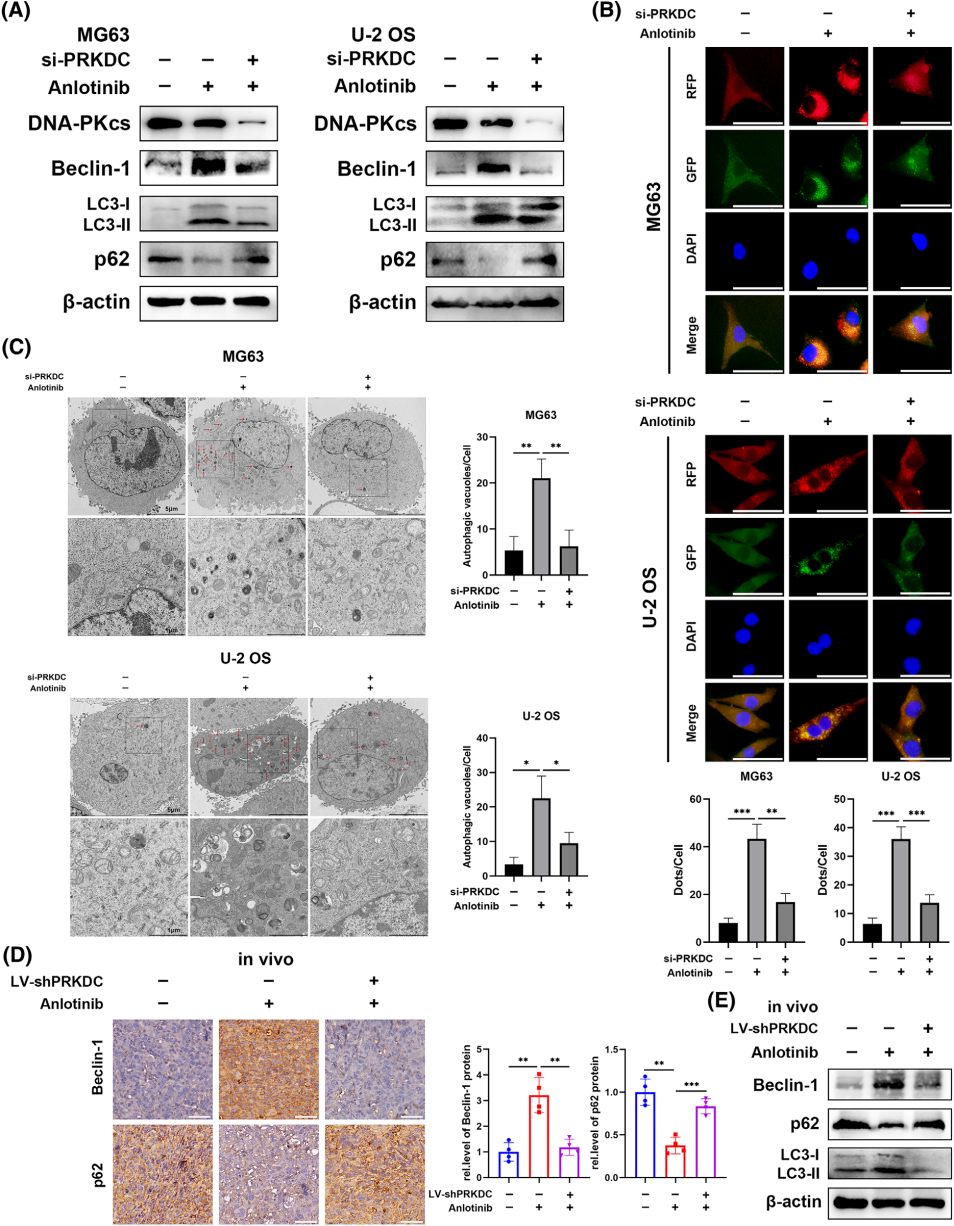
DNA‐PKcs knockdown suppresses anlotinib‐induced autophagy in vitro and in vivo. MG63 and U‐2 OS cells were transfected with PRKDC siRNA and subsequently treated with anlotinib for 24 h. (A) Western blot analysis was used to detect the expression of DNA‐PKcs and autophagy‐related proteins in MG63 and U‐2 OS cells. (B) MG63 and U‐2 OS cells were infected with RFP‐GFP‐LC3 lentivirus and treated with si‐NC or si‐PRKDC and anlotinib. A fluorescence microscope was used to image autophagosomes and autolysosomes in the cells, scale bar = 50µm. (C) Representative images of autophagosomes in MG63 and U‐2 OS cells obtained via transmission electron microscopy, scale bar = 5µm (upper), 1µm (below, enlarged). (D) IHC staining images of Beclin‐1 and p62 in xenograft tumours in the CTR, anlotinib, and LV‐shPRKDC+anlotinib groups, scale bar = 50µm. (E) Western blot analysis was used to assess Beclin‐1, p62 and LC3‐II/I protein expression in the xenograft tumours of each group. *n* = 3. Data are presented as mean ± SD, *n* = 3. *p < .05; **p < .01; ***p < .001 (Student's *t*‐test for two‐group comparisons, one‐way ANOVA with Bonferroni post‐tests for multiple group comparisons).

A previous study showed that anlotinib triggers a defensive response against autophagy in cancer cells, and blocking this process boosts the effectiveness of anlotinib against cancer cells.^6^ Therefore, we hypothesised that DNA‐PKcs might influence anlotinib‐induced autophagic responses. As expected, western blot analysis revealed a significant decrease in Beclin‐1 expression and conversion of LC3‐I to LC3‐II, along with an increase in p62 expression in MG63 and U‐2 OS cells following treatment with anlotinib and transfection with PRKDC siRNA (Figures 2A and S1G). We evaluated the effects of si‐PRKDC treatment alone on autophagy in osteosarcoma cells. As shown in Figure S1H, si‐PRKDC treatment notably enhanced autophagy in these cells. Given the specificity of the cellular response to distinct stimuli, we speculated that PRKDC knockdown may also modulate autophagy through alternative mechanisms. As this aspect is transcend the current research, it will be investigated in future studies. Subsequently, we co‐transfected MG63 and U‐2 OS cells with PRKDC siRNA or control siRNA and RFP‐GFP‐LC3 lentivirus. Notably, the numbers of anlotinib‐induced autophagosomes and autolysosomes decreased in DNA‐PKcs‐knockdown osteosarcoma cells (Figure 2B). Correspondingly, electron microscopy exhibited that anlotinib therapy remarkably raised the quantity of autophagic vacuoles with intact membranes in the cytoplasm, whereas the simultaneous knockdown of PRKDC notably reduced the number of autophagic vacuoles (Figure 2C, indicated by red arrows). Furthermore, in the MG63 cell‐mediated xenograft model, IHC staining showed a notable reduction in Beclin‐1 levels and an increase in p62 levels in specimens from the cohort receiving anlotinib and PRKDC shRNA transfection compared to those exposed to control lentivirus and anlotinib (Figure 2D). Additionally, examination of xenograft tissues by western blotting showed a notable reduction in Beclin‐1 expression, anlotinib‐induced LC3‐I to LC3‐II conversion, and a remarkable induction of p62 expression upon silencing of DNA‐PKcs (Figure 2E).

**FIGURE 3:**
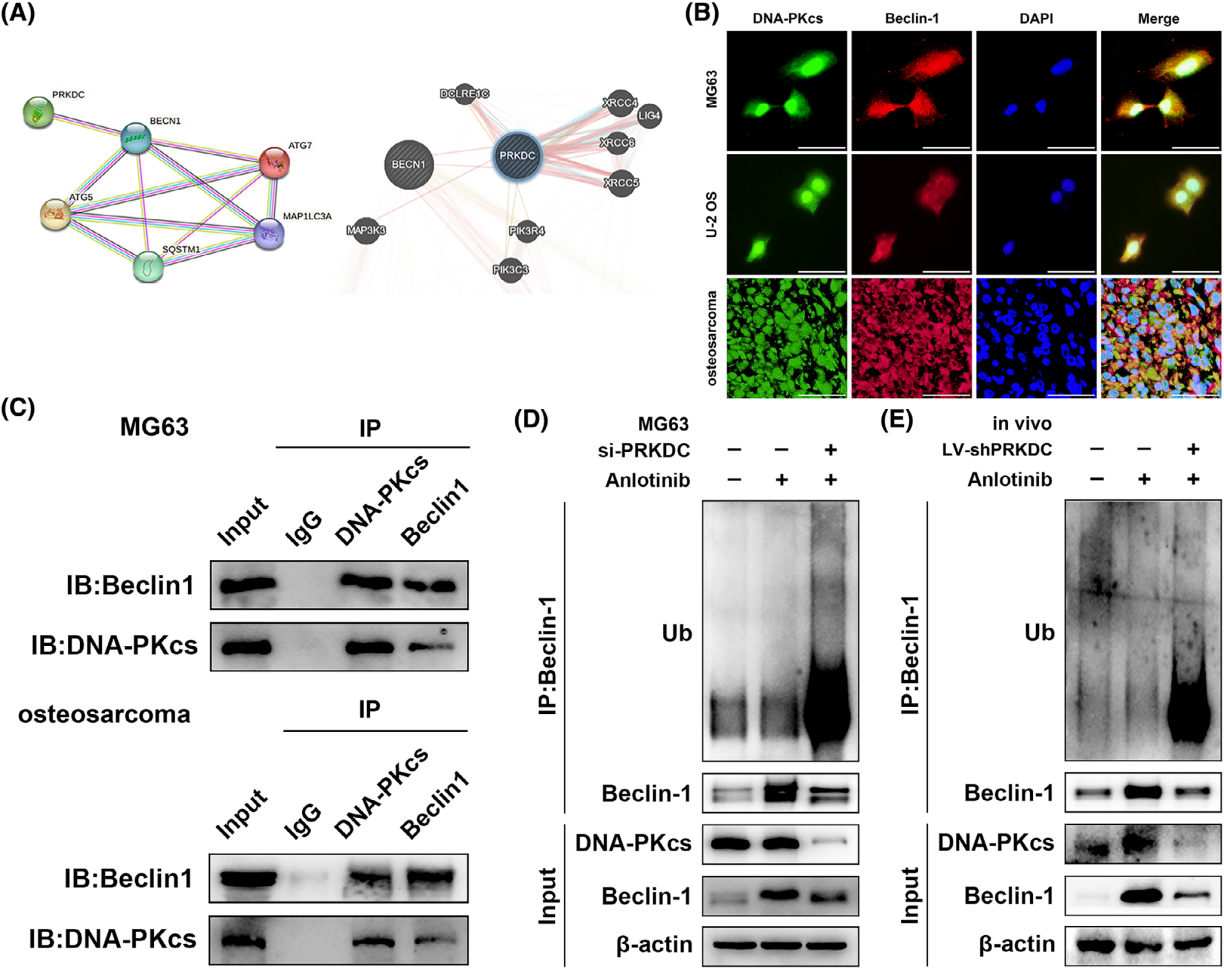
DNA‐PKcs interacts with Beclin‐1 and reduces its ubiquitination. (A) Bioinformatics analysis predicts an interaction between DNA‐PKcs and Beclin‐1 (left: STRING, https://string‐db.org/, right: GeneMANIA, https://genemania.org/). (B) Immunofluorescence measured the co‐localisation of DNA‐PKcs (green) and Beclin‐1 (red) in MG63, U‐2 OS cells, and human osteosarcoma tissues. Nuclei were stained with DAPI. Scale bar = 50µm. (C) Co‐IP assay examined the interaction between DNA‐PKcs and Beclin‐1 in MG63 cells and human osteosarcoma tissues. (D) Beclin‐1 IP and western blot analysis assessed Beclin‐1 ubiquitination in MG63 cells following DNA‐PKcs knockdown. (E) Xenograft tumour lysates from each group were immunoprecipitated with anti‐Beclin‐1, and western blotting was used to detect Beclin‐1 ubiquitination. *n* = 3.

To investigate the molecular mechanism by which the downregulation of DNA‐PKcs influences anlotinib‐induced autophagy, we performed bioinformatics analysis using the STRING, GeneMANIA, and HitPredict databases to assess the correlation between DNA‐PKcs and Beclin‐1, p62, ATG5, ATG7, and MAP1LC3A, which suggested a potential interaction between DNA‐PKcs and Beclin‐1 (Figure 3A and Supplementary Material). As presented in Figure S1I, treatment with si‐PRKDC+ anlotinib did not reduce the mRNA level of Beclin‐1 relative to that in the si‐NC+ anlotinib group, indicating that the connection between DNA‐PKcs and Beclin‐1 was not manifested at the mRNA level. Additionally, IF staining demonstrated the co‐localisation of DNA‐PKcs with Beclin‐1 in MG63 cells, U‐2 OS cells, as well as in human osteosarcoma tissues (Figure 3B). Remarkably, co‐IP revealed that Beclin‐1 was successfully precipitated by the DNA‐PKcs antibody, whereas DNA‐PKcs was pulled down by the anti‐Beclin‐1 antibody in MG63 cells and human osteosarcoma tissues, providing evidence for the interplay between DNA‐PKcs and Beclin‐1 (Figure 3C). Given the observed impact of DNA‐PKcs on Beclin‐1 expression at the protein level, we sought to confirm whether DNA‐PKcs regulates the ubiquitination and breakdown processes of Beclin‐1. As expected, Beclin‐1 ubiquitination was effectively increased, and its protein expression level was decreased in the anlotinib‐treated group with DNA‐PKcs knockdown, while MG132 treatment partially reduced the degradation of Beclin‐1 (Figures 3D and S1J). Similarly, in the MG63 cell‐mediated xenograft model, a noticeable enhancement was observed in Beclin‐1 ubiquitination and degradation in the presence of shPRKDC along with anlotinib compared to tumours treated with control lentivirus and anlotinib (Figure 3E).

As a result, the findings suggest that DNA‐PKcs modulate autophagy by interacting with Beclin‐1 and regulating its ubiquitination.

### 3.3 METTL3 involved in the anlotinib resistance through mediating the m^6^A modification of PRKDC and promoting its expression

**FIGURE 4:**
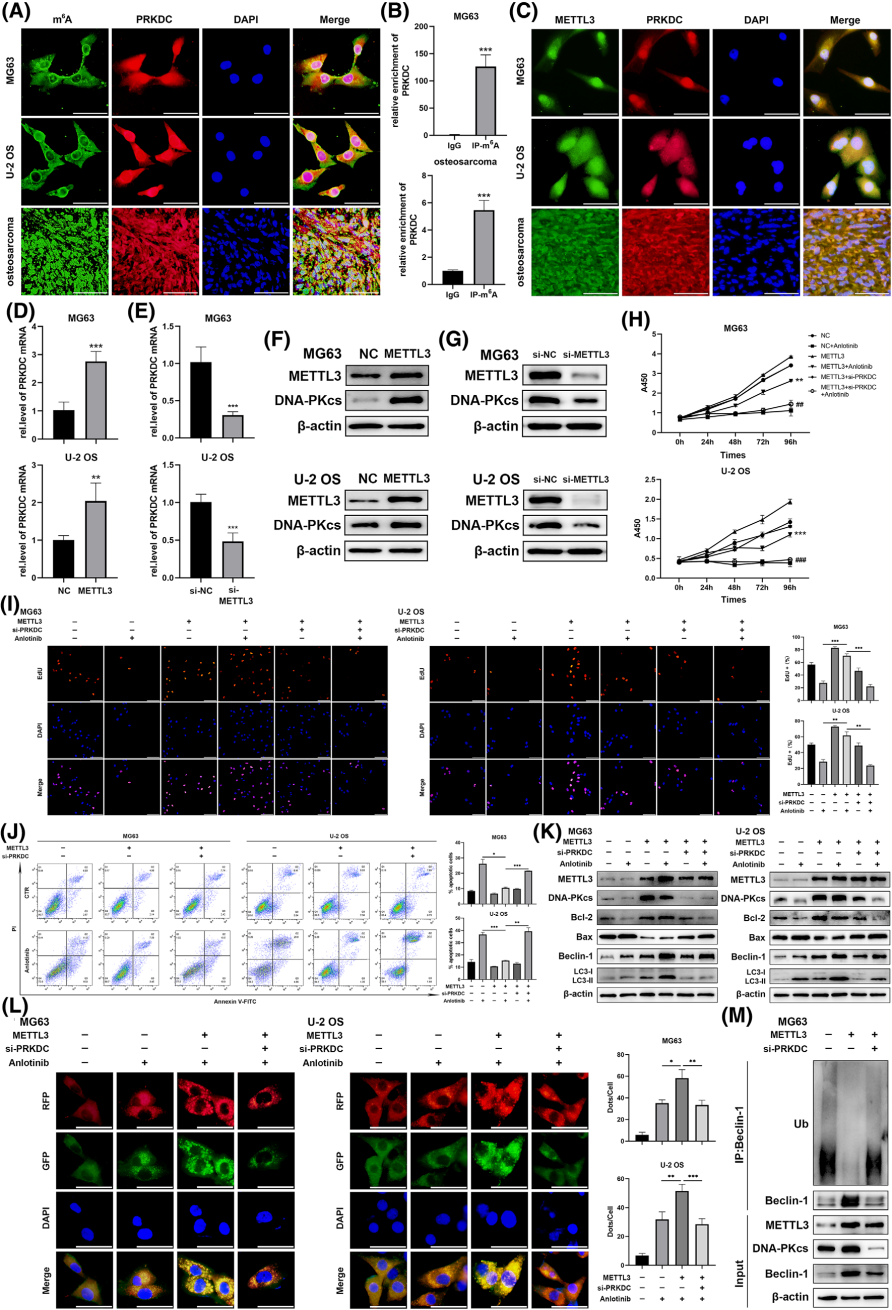
METTL3 fosters anlotinib resistance by mediating PRKDC m^6^A modification and expression. (A) RNA FISH‐immunofluorescence showed the co‐localisation of m^6^A (green) and PRKDC mRNA (red) in MG63, U‐2 OS cells, and human osteosarcoma tissues, scale bar = 50µm. (B) The m^6^A modification of PRKDC was determined in MG63 and human osteosarcoma tissues using MeRIP‐qPCR. (C) RNA FISH‐immunofluorescence confirmed the co‐localisation of METTL3 (green) and PRKDC mRNA (red) in MG63, U‐2 OS cells, and human osteosarcoma tissues, scale bar = 50µm. (D, E) The qRT‐PCR assay assessed PRKDC mRNA expression in MG63 and U‐2 OS cells with METTL3 overexpression or knockdown. (F, G) Western blot analysis measured METTL3 and DNA‐PKcs protein levels in MG63 and U‐2 OS cells following transfection with METTL3 plasmid or siRNA. MG63 and U‐2 OS cells were co‐transfected with METTL3 plasmid and PRKDC siRNA or NC siRNA, then treated with anlotinib. (H) The CCK8 assay assessed the viability of MG63 and U‐2 OS cells, **p < .01; ***p < .001 (VS NC+Anlotinib), ^##^ *p* < .01; ^###^ *p* < .001 (VS METTL3+Anlotinib). (I) EdU staining evaluated MG63 and U‐2 OS cell proliferation after treated with anlotinib for 24 h, scale bar = 100µm. (J) Annexin V‐FITC/PI staining followed by flow cytometry was used to measure the percentage of apoptotic cells. (K) Western blot analysis assessed the protein levels of METTL3, DNA‐PKcs, Bcl‐2, Bax, Beclin‐1, and LC3‐II/I following the treatment with anlotinib for 24 h. (L) Fluorescence microscopy visualised autophagosomes and autolysosomes in cells stably infected with RFP‐GFP‐LC3 lentivirus after treated with anlotinib for 24 h, scale bar = 50µm. (M) Beclin‐1 IP and western blotting assessed Beclin‐1 ubiquitination in MG63 cells after co‐transfection with METTL3 plasmid and PRKDC siRNA or NC siRNA. Data are presented as mean ± SD, *n* = 3. **p < .01; ***p < .001 (Student's *t*‐test for two‐group comparisons, one‐way ANOVA with Bonferroni post‐tests for multiple group comparisons).

Our study elucidated the elevation of DNA‐PKcs in response to anlotinib treatment, establishing its correlation with osteosarcoma sensitivity to anlotinib. However, the reason for this increase is not yet fully understood. One of the key modifications found in mRNA, m^6^A modification, is an important player in diverse activities, like mRNA splicing, transportation, stabilisation, and degradation.^21^, ^30^ Therefore, we hypothesised that the PRKDC mRNA undergoes m^6^A modification. To test this hypothesis, we performed m^6^A IF assays and PRKDC mRNA FISH. As depicted in Figure 4A, m^6^A and PRKDC exhibited co‐localisation in MG63 and U‐2 OS cells and in human osteosarcoma tissues. Furthermore, substantial enrichment of PRKDC was observed in m^6^A immunoprecipitation (IP) but not in IgG IP (Figure 4B), supporting the presence of m^6^A methylation in PRKDC mRNA. Remarkably, Figure S2A shows that following anlotinib treatment, the m^6^A level of PRKDC mRNA was significantly enhanced, indicating that the methylation of PRKDC mRNA was influenced by anlotinib treatment.

The m^6^A on RNA involves writers (m^6^A methyltransferases, like METTL3, METTL14, and WTAP), (m^6^A demethylases, such as FTO and ALKBH5),^21^, ^30^, ^31^ etc. To recognise the key regulators of m^6^A modification in PRKDC mRNA regulation, we conducted bioinformatics predictions. We performed a correlation analysis between PRKDC and METTL3, METTL14, WTAP, FTO, and ALKBH5 using the GeneMANIA platform. As illustrated in Figure S2B, PRKDCs interacted between PRKDC and METTL3 or METTL14. Furthermore, qRT‐PCR assays revealed a significant increase in METTL3 expression following anlotinib treatment in MG63 and U‐2 OS cells, but not in METTL14 expression (Figure S2C). Similarly, western blot analysis showed that METTL3 protein levels increased notably after 48, 72, and 96 h of anlotinib treatment in MG63 and U‐2 OS cells (Figure S2D). FISH‐IF co‐localisation assay confirmed similar distributions of METTL3 protein and PRKDC mRNA in MG63, U‐2 OS cells, and human osteosarcoma tissues (Figure 4C). Given its crucial role in m^6^A modification, we hypothesised that METTL3 serves as the primary regulator of PRKDC m^6^A modification. To test this hypothesis, we transfected METTL3 plasmids and siRNA into MG63 and U‐2 OS cells and confirmed their transfection efficiency (Figure S2E and F). Remarkably, qRT‐PCR and western blot assays showed a significant elevation in PRKDC mRNA and DNA‐PKcs protein levels upon METTL3 overexpression, whereas suppression of METTL3 resulted in the depletion of PRKDC mRNA and DNA‐PKcs protein expression (Figures 4D–G and S2G and H).

To explore the potential involvement of METTL3 and DNA‐PKcs in the response of osteosarcoma cells, CCK8 and EdU staining assays were performed. Transfection with the METTL3 plasmid significantly attenuated anlotinib‐induced reduction in the proliferation of MG63 and U‐2 OS cells, and this effect that was reversed by PRKDC siRNA (Figure 4H and I). Flow cytometry demonstrated that METTL3 overexpression significantly decreased anlotinib‐induced apoptosis, which was rescued by PRKDC siRNA (Figure 4J). Similarly, following METTL3 plasmid transfection in the group treated with anlotinib, there was a rise in Bcl‐2 expression and a reduction in Bax expression in MG63 and U‐2 OS cells, and PRKDC siRNA attenuated this effect (Figure 4K). Additionally, we examined the anlotinib‐induced autophagic responses after transfection with METTL3 and PRKDC siRNAs. The level of Beclin‐1 and the transformation of LC3‐I to LC3‐II induced by anlotinib were remarkably improved among METTL3 overexpressing cells but were reversed by PRKDC siRNA, as decided by western blot analysis (Figure 4K). Additionally, the METTL3 overexpression group in MG63 and U‐2 OS cells transfected with RFP‐GFP‐LC3 lentivirus exhibited a notable increase in anlotinib‐induced autophagosomes and autolysosomes, which was rescued by PRKDC siRNA transfection (Figure 4L). Notably, METTL3 overexpression markedly reduced Beclin‐1 ubiquitination and increased its protein expression. These effects were reversed by DNA‐PKcs knockdown (Figure 4M). These findings suggested that METTL3 promotes anlotinib resistance in osteosarcoma by regulating the expression of DNA‐PKcs.

### 3.4 METTL3 increased PRKDC m^6^A methylation

**FIGURE 5:**
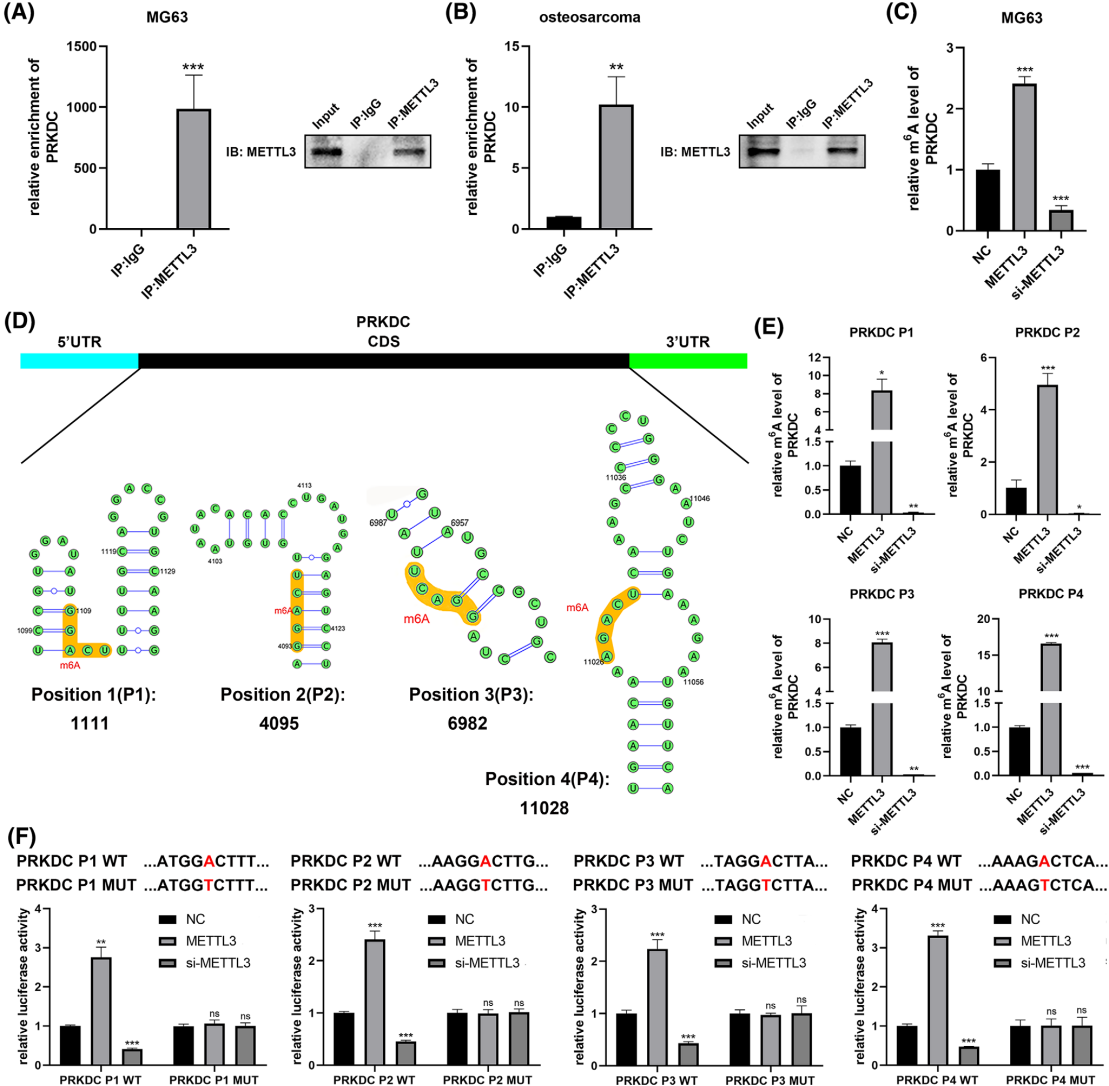
METTL3 regulates the m^6^A modification of PRKDC. (A, B) RNA immunoprecipitation (RIP) targeting METTL3 was conducted in MG63 cells and human osteosarcoma tissues. The PRKDC mRNA levels were measured via qRT‐PCR, and METTL3 protein expression was assessed by western blotting. (C) MeRIP‐qPCR was used to detect PRKDC m^6^A modification levels in MG63 cells transfected with METTL3 plasmid or siRNA. (D) Four potential m^6^A positions of PRKDC mRNA were predicted using the SRAMP database (http://www.cuilab.cn/sramp). (E) MeRIP‐qPCR measured the m^6^A modification levels in four specific PRKDC regions in MG63 cells transfected with METTL3 plasmid or siRNA. (F) Wild‐type (WT) and mutant (MUT) sequences of PRKDC mRNA m^6^A positions were designed, and their luciferase activities were measured in MG63 cells with METTL3 overexpression or knockdown. Data are presented as mean ± SD, *n* = 3. *p < .05; **p < .01; ***p < .001 (Student's *t*‐test for two‐group comparisons, one‐way ANOVA with Bonferroni post‐tests for multiple group comparisons).

Subsequently, we investigated the potential mechanism through which METTL3 regulates DNA‐PKcs expression. Remarkably, RNA immunoprecipitation (RIP) assays conducted in MG63 cells and human osteosarcoma tissues revealed significant enrichment of PRKDC in the METTL3 immunoprecipitation group, however, not in IgG control group (Figure 5A and B). Similarly, anlotinib treatment significantly enhanced PRKDC enrichment in the METTL3 RIP assay (Figure S2I). Additionally, MeRIP‐qPCR demonstrated that METTL3 overexpression markedly increased m^6^A methylation in PRKDC, whereas METTL3 knockdown significantly reduced PRKDC methylation (Figure 5C). Given that m^6^A modifications typically occur in the RRAC consensus motif, a sequence‐based predictor known as SRAMP was used to identify potential m^6^A sites within the coding region (CDS) of the PRKDC mRNA (Figures 5D and S2J). MeRIP‐qPCR assays based on the predicted m^6^A sites revealed four potential positions that were significantly enriched by the m^6^A antibody, and their methylation levels substantially increased upon METTL3 overexpression but decreased upon METTL3 knockdown, indicating that METTL3 methylates these sites in PRKDC mRNA (Figure 5E). Furthermore, we constructed four double‐luciferase reporter plasmids containing wild‐type or mutant sequences of the predicted m^6^A sites in PRKDC mRNA before the firefly luciferase‐coding region (Figure 5F). Figure S2K confirms that the transfection of wild‐type and mutant reporter plasmids did not remarkably impact the expressions of PRKDC. Intriguingly, the luciferase reporter assays manifested that the luciferase activity of the wild‐type reporters was remarkably increased with METTL3 overexpression compared to the mutant‐type reporters at the four predicted positions in PRKDC. Conversely, when METTL3 was knocked down, the luciferase activity of wild‐type reporters decreased, suggesting a reduction in PRKDC methylation due to the mutation of the m^6^A site (Figure 5F).

Accordingly, it indicates that METTL3 is a key player in enhancing the m^6^A modification of PRKDC mRNA, increasing its expression levels.

### 3.5 YTHDF1 recognised PRKDC m^6^A modification and regulated the sensitivity of osteosarcoma to anlotinib to assist with METTL3

**FIGURE 6:**
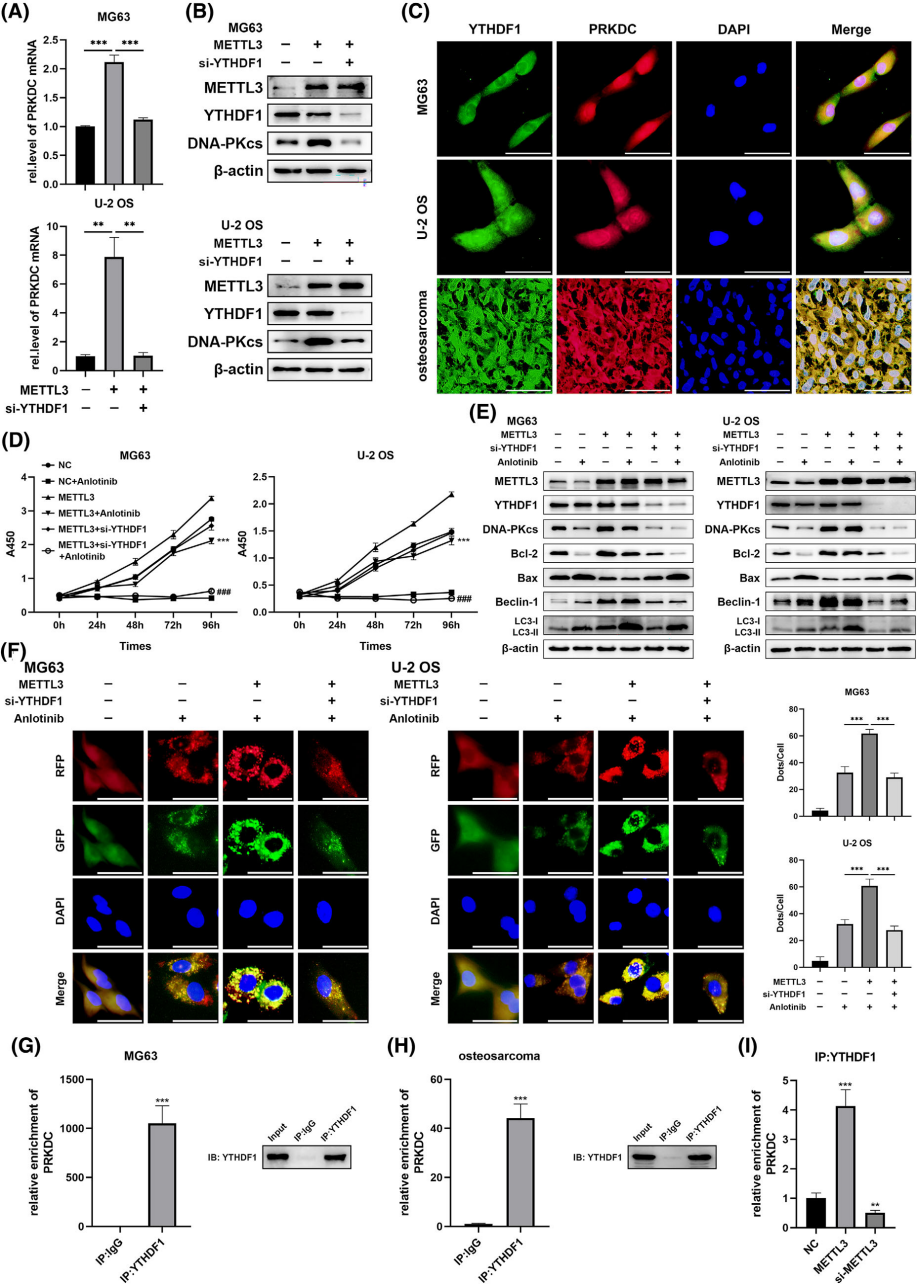
YTHDF1 recognises PRKDC m^6^A modification and enhances anlotinib resistance. (A) The qRT‐PCR assay determined PRKDC mRNA expression in MG63 and U‐2 OS cells co‐transfected with METTL3 plasmid and YTHDF1 siRNA. (B) Western blotting was used to measure METTL3, YTHDF1, and DNA‐PKcs protein levels in MG63 and U‐2 OS cells co‐transfected with the METTL3 plasmid and YTHDF1 siRNA or NC siRNA. (C) RNA FISH‐immunofluorescence confirmed the co‐localisation of YTHDF1 (green) and PRKDC mRNA (red) in MG63, U‐2 OS cells, and human osteosarcoma tissues, scale bar = 50µm. MG63 and U‐2 OS cells were co‐transfected with the METTL3 plasmid and YTHDF1 siRNA or NC siRNA, then treated with anlotinib. (D) The CCK8 assay measured MG63 and U‐2 OS cell viability, ***p < .001 (VS NC+Anlotinib), ^###^ *p* < .001 (VS METTL3+Anlotinib). (E) Western blotting assessed the protein levels of METTL3, YTHDF1, DNA‐PKcs, Bcl‐2, Bax, Beclin‐1, and LC3‐II/I after treated with anlotinib for 24 h. (F) Fluorescence microscopy imaged autophagosomes and autolysosomes in cells stably infected with the RFP‐GFP‐LC3 lentivirus following the treatment with anlotinib for 24 h, scale bar = 50µm. (G, H) RIP for YTHDF1 was conducted in MG63 cells and human osteosarcoma tissues. PRKDC mRNA levels were measured via qRT‐PCR, and YTHDF1 protein expression was detected using western blotting. (I) RIP‐qPCR analysed the interaction between YTHDF1 and PRKDC mRNA in MG63 cells with METTL3 overexpression or knockdown. Data are presented as mean ± SD, *n* = 3. **p < .01; ***p < .001 (Student's *t*‐test for two‐group comparisons, one‐way ANOVA with Bonferroni post‐tests for multiple group comparisons).

Previous studies have indicated that m^6^A‐mediated function depends on ‘readers’, such as YTHDF1, YTHDF2, YTHDF3, or YTHDC1.^21^, ^30^ To investigate the regulatory mechanism of METTL3 on PRKDC, we performed bioinformatics analysis to identify which ‘reader’ may be involved in regulating PRKDC m^6^A modification. We performed correlation analysis between PRKDC and YTHDF1, YTHDF2, YTHDF3, and YTHDC1 using the GeneMANIA database. As shown in Figure S3A, PRKDC may interact with YTHDF1 or YTHDF2. YTHDF1 promotes the translation of methylated transcripts, whereas YTHDF2 predominantly accelerates mRNA decay.^21^, ^32^ Subsequently, we transfected MG63 and U‐2 OS cells with the METTL3 plasmid and either YTHDF1 or YTHDF2 siRNA, and the efficacy of siRNA transfection was confirmed (Figure S3B and C). Notably, qRT‐PCR and western blotting assays manifested that YTHDF1, but not YTHDF2, significantly reversed METTL3‐mediated upregulation of PRKDC mRNA and DNA‐PKcs proteins in MG63 and U‐2 OS cells (Figures 6A and B and S3D). Furthermore, FISH‐IF assays confirmed the co‐localisation of YTHDF1 protein and PRKDC mRNA in MG63 and U‐2 OS cells as well as in human osteosarcoma tissues (Figure 6C). Furthermore, YTHDF1 RIP assays revealed that anlotinib treatment significantly enhanced PRKDC enrichment in YTHDF1 cells (Figure S3E). Hence, we hypothesised that YTHDF1 might serve as a ‘reader’ of PRKDC m^6^A methylation, thereby affecting the sensibility of osteosarcoma cells to anlotinib.

To validate this hypothesis, the METTL3 plasmid and YTHDF1 siRNA were transfected into MG63 and U‐2 OS cells, respectively, followed by treatment with anlotinib. Subsequently, experiments, including the CCK8 assay and western blotting, were conducted to validate the results. The results indicated that METTL3 overexpression significantly attenuated anlotinib‐induced reduction in cell proliferation and changes in apoptosis‐related proteins (Bcl‐2 and Bax), whereas YTHDF1 knockdown reversed these effects (Figure 6D and E). Moreover, western blot analysis showed that the alterations triggered by METTL3 overexpression in Beclin‐1 expression and the transformation of LC3‐I to LC3‐II were restored through the YTHDF1 knockdown (Figure 6E). Similarly, significant augmentation in anlotinib‐induced autophagosomes and autolysosomes was observed in MG63 and U‐2 OS cells transfected with RFP‐GFP‐LC3 lentivirus when METTL3 was overexpressed, which was counteracted by YTHDF1 siRNA transfection (Figure 6F). We investigated whether YTHDF1 recognised PRKDC m^6^A modifications. RIP assays demonstrated that YTHDF1 bound directly to PRKDC mRNA in MG63 cells and human osteosarcoma tissues (Figure 6G and H). Surprisingly, the interaction between YTHDF1 and PRKDC mRNA was enhanced in METTL3 overexpressing MG63 cells, whereas the METTL3 knockdown reduced this interaction (Figure 6I). In addition, MeRIP‐qPCR assays based on four potential m^6^A positions revealed that methylation levels increased substantially upon METTL3 overexpression and were reversed by YTHDF1 knockdown, indicating that YTHDF1 binds to these sites in the PRKDC mRNA (Figure S3F).

Altogether, these results confirm that YTHDF1 regulates METTL3‐mediated PRKDC m^6^A modification, contributing to the maintenance of anlotinib resistance in osteosarcoma.

### 3.6 METTL3 promoted osteosarcoma resistant to anlotinib via inducing PRKDC m^6^A modification in vivo

**FIGURE 7:**
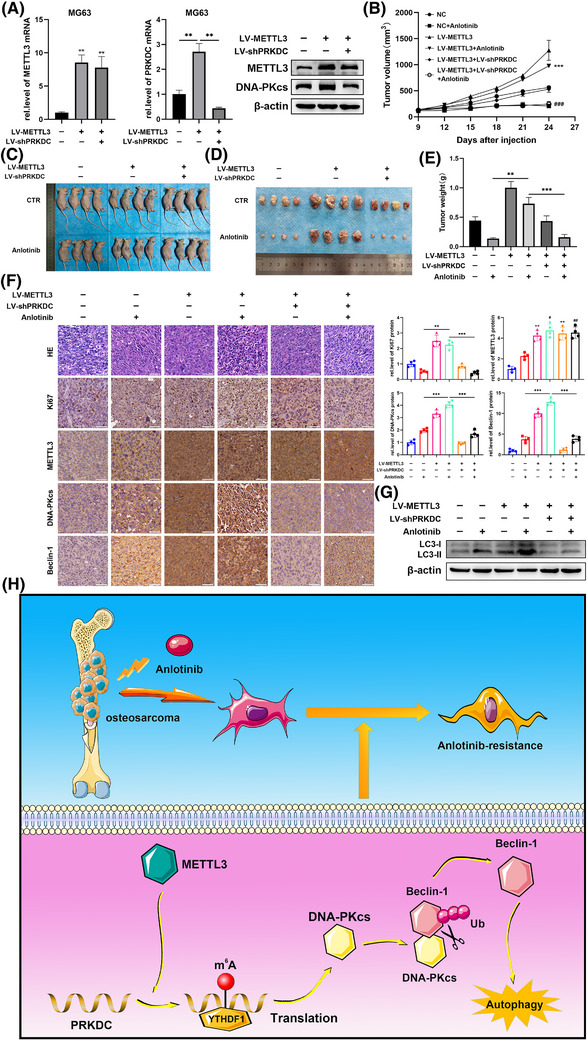
METTL3 sustains anlotinib resistance by regulating PRKDC m^6^A modification in vivo. (A) PRKDC and METTL3 mRNA levels, as well as METTL3 and DNA‐PKcs protein levels, were assessed via qRT‐PCR and western blotting in control lentivirus, METTL3 lentivirus alone, or METTL3 lentivirus combined with PRKDC shRNA lentivirus‐stable MG63 cell infections. Stably expressing MG63 cells were injected into nude mice and treated with anlotinib. (B) Tumour volume was measured every 3 days, ***p < .001 (VS NC+Anlotinib), **^###^** *p* < .001 (VS LV‐METTL3+Anlotinib). (C) Mice were sacrificed, and representative images of MG63 tumours were captured at the termination of experiment. (D) Representative images of xenograft tumours. (E) MG63 xenograft tumour weights were measured. (F) H&E and IHC staining of Ki67, METTL3, DNA‐PKcs, and Beclin‐1 in tumour xenografts from each group, scale bar = 50µm, **p < .01 (VS CTR), **^#^** *p* < .05, ^##^ *p* < .01 (VS Anlotinib). (G) Western blot analysis determined LC3‐II/I protein expression in xenograft tumours from each group. (H) Graphic abstract illustrating how METTL3 regulates anlotinib resistance in osteosarcoma through PRKDC mRNA m^6^A modification. Data are presented as mean ± SD, *n* = 4. **p < .01; ***p < .001 (one‐way ANOVA with Bonferroni post‐tests).

To further reinforce the link between METTL3, DNA‐PKcs, and anlotinib sensitivity as established previously, and to discuss its potential clinical implications, tumour xenograft models were constructed among nude mice through MG63 cells consistently transfected with METTL3 lentivirus or control lentivirus and concurrently with PRKDC shRNA lentivirus or control shRNA lentivirus. Figures 7A and S3G show the effectiveness of shRNA lentivirus transfection. When palpable tumours were observed, the mice were administered either the control vehicle or anlotinib. The results presented in Figure 7B–E demonstrate that METTL3 overexpression significantly bolstered tumour xenograft resistance to anlotinib‐induced responses, an effect that was negated by co‐transfection with PRKDC shRNA lentivirus. Consistently, IHC staining of xenograft tissues revealed an increase in Ki67 expression in the anlotinib group following transfection with the METTL3 lentivirus compared to that in tumours in the anlotinib group transfected with the control lentivirus, whereas this expression was reduced in tissues co‐transfected with the shPRKDC lentivirus (Figure 7F). Additionally, a rise in Beclin‐1 expression and shift from LC3‐I to LC3‐II induced by anlotinib was notably examined through IHC staining and western blotting assays in tumours transfected with the METTL3 lentivirus; however, this effect was abolished by co‐transfection with the PRKDC shRNA lentivirus (Figure 7F and G).

Overall, our results showed that DNA‐PKcs interact with Beclin‐1, thereby enhancing the autophagy response and ultimately leading to anlotinib resistance in osteosarcoma. Elevated levels of PRKDC m^6^A modification, mediated by METTL3/YTHDF1, contributed to increased DNA‐PKcs protein expression (Figure 7H).

## 4 DISCUSSION

Anlotinib is new oral inhibitor that targets various receptor tyrosine kinases (RTKs) to treat osteosarcoma, especially after traditional chemotherapy is ineffective or during advanced tumour stages.^12^, ^17^, ^33^ Nevertheless, developing acquired resistance to osteosarcoma therapy is a significant obstacle, underscoring the immediate necessity for clinical exploration of the underlying mechanisms. In this study, we made several key observations. Initially, DNA‐PKcs knockdown enhanced osteosarcoma sensitivity to anlotinib. Subsequently, DNA‐PKcs modulated anlotinib‐induced protective autophagy by interacting with Beclin‐1 and regulating its ubiquitination. Additionally, we explored whether the m^6^A modification of PRKDC mRNA induced by METTL3 contributed to anlotinib resistance in osteosarcoma. Furthermore, m^6^A methylation of PRKDC mRNA, which is recognised by YTHDF1 amplifies the expression of DNA‐PKcs. Our findings confirm the significance and efficacy of targeting METTL3‐mediated m^6^A modification and DNA‐PKcs in overcoming drug resistance in osteosarcoma, potentially offering a novel therapeutic strategy for this challenging malignancy.

DNA‐PKcs, forming a complex with Ku70 and Ku80, participate in the repair of DNA injury through non‐homologous end‐joining (NHEJ), leading to adverse clinical outcomes in a variety of cancer types.^27^, ^34^, ^35^, ^36^ Prior research has unveiled that suppressing DNA‐PKcs may enhance telomere dysfunction and induce cell death in glioblastoma cells.^37^ Furthermore, Liu et al. demonstrated that an Hsp90 inhibitor enhanced hyperthermia‐induced DNA damage by downregulating DNA‐PKcs.^38^ Collectively, these studies underscore the importance of targeting DNA‐PKcs as a critical strategy for cancer therapy. Notably, our study revealed that anlotinib treatment for over 48 h increased DNA‐PKcs expression in osteosarcoma cells, suggesting its potential relevance to the sensitivity of osteosarcoma to anlotinib. In addition, following treatment through anlotinib for 1 day, the expression of DNA‐PKcs moderately decreased. This may be an initial stress response that leads to a temporary decrease in DNA‐PKcs levels, possibly as part of a regulatory mechanism for managing DNA damage or cellular stress; however, this requires further investigation. Our previous study showed that suppression of DNA‐PKcs sensitises osteosarcoma cells to chemotherapeutic agents by reducing P‐gp expression.^27^ Consistent with this finding, Fang et al. demonstrated that DNA‐PKcs stabilised SOX2 to modulate glioblastoma sensitivity to radiation.^39^ However, prior to our study, the role of DNA‐PKcs in anlotinib resistance in osteosarcoma remains unclear. Remarkably, our findings revealed a pioneering revelation that depletion of DNA‐PKcs strengthened the reactions triggered by anlotinib in osteosarcoma models, both in vitro and in vivo. However, the potential mechanisms of the effects of DNA‐PKcs on anlotinib‐sensitivity in osteosarcoma warrant further investigation.

Several studies have elucidated the complex role of autophagy, which can either inhibit or promote cell apoptosis, depending on the context.^40^, ^41^, ^42^ Most studies indicate that autophagy is primarily a stress adaptation mechanism that reduces apoptosis and promotes cell survival.^42^, ^43^, ^44^ In particular, clinical studies have revealed that autophagy inhibitors such as chloroquine and hydroxychloroquine may enhance the antitumour effects of chemotherapy and radiation.^45^ Ma et al. demonstrated that sensitisation of gastric cancer cells to vascular endothelial growth factor receptor‐2 (VEGFR‐2) tyrosine kinase suppressors through autophagy inhibition was mediated by the downregulation of circRACGAP1.^46^ Furthermore, blocking autophagy triggered by anlotinib has been shown to halt the progression of tumours among diverse categories of carcinomas, like NSCLC, anaplastic thyroid cancer, and osteosarcoma.^6^, ^47^, ^48^ Therefore, we hypothesised that DNA‐PKcs may regulate protective autophagy induced by anlotinib. A previous study noted that autophagy triggered by genotoxic stress could be controlled by the activation of DNA‐PKcs and PARP‐1 by ATM.^49^ Similarly, Puustinen et al. demonstrated that PRKDC depletion inhibited DNA damage‐induced autophagic flux by regulating AMPK phosphorylation.^50^ Remarkably, we observed a reduction in autophagy after treatment with anlotinib in DNA‐PKcs knockdown osteosarcoma cells both in vivo and in vitro. Notably, our study identified an increase in autophagy following PRKDC silencing. The effect of the PRKDC knockdown on autophagy remains a topic of considerable debate, with most studies suggesting that PRKDC depletion suppresses drug‐induced autophagy. Some studies have demonstrated that inhibiting DNA‐PKcs expression enhances the sensitivity of glioma cells to radiotherapy.^51^, ^52^ However, in agreement with our findings, PRKDC siRNA was shown to significantly inhibit etoposide‐induced autophagy,^50^ whereas DNA‐PKcs was implicated in capsaicin‐induced protective autophagy.^49^ This discrepancy may arise from the distinct roles that DNA‐PKcs play in regulating autophagy in response to different stimuli, which will be further explored in future studies. Subsequent bioinformatic predictions suggested that DNA‐PKcs might interact with Beclin‐1. The interaction between DNA‐PKcs and Beclin‐1 was further confirmed by IF staining and co‐IP assays. Additionally, we found for the first time that DNA‐PKcs regulate Beclin‐1 ubiquitination and promote its stability. Overall, the significant role of DNA‐PKcs in adjusting the responsiveness of osteosarcoma cells to anlotinib was highlighted. However, further investigation is required to elucidate the mechanisms contributing to the enhanced expression of DNA‐PKcs induced by anlotinib.

Notably, we observed m^6^A modifications in PRKDC mRNA in MG63 and U‐2 OS cells and in human osteosarcoma tissues. As a well‐established epigenetic regulatory mechanism, m^6^A significantly affects RNA production, stability, translation, and interactions.^21^, ^30^ The essential contribution of m^6^A modifications to tumour progression has been highlighted in previous research. Xu et al. demonstrated that circRNA‐SORE, which maintains sorafenib resistance in hepatocellular cancer, is stabilised by m6A modifications.^30^ In addition, METTL3‐ and METTL14‐mediated m^6^A modifications of TRIM7 regulate osteosarcoma metastasis and chemoresistance.^53^ Furthermore, the enzymes responsible for m^6^A methylation, known as ‘writers’ and ‘erasers’, play pivotal roles in this process. METTL3, an m^6^A methyltransferase, regulates the m^6^A level LEF1, promoting osteosarcoma progression.^54^ Similarly, Yuan et al. demonstrated that the demethylase ALKBH5 downregulates YAP expression, leading to tumour inhibition in osteosarcoma.^55^ Our findings elucidate, for the first time, the regulation of DNA‐PKcs expression by METTL3, thereby enhancing osteosarcoma resistance to anlotinib. Moreover, we observed an interaction between METTL3 and PRKDC mRNA. METTL3 overexpression increased PRKDC m^6^A methylation, whereas METTL3 knockdown abrogated this effect. However, the downstream ‘reader’ of PRKDC m^6^A modification in osteosarcoma remains elusive and necessitates further investigation.

m^6^A modification exerts its biological effects by interacting with downstream readers.^21^ For instance, YTHDF1 can engage with translation initiation machinery to mediate the translation of recognised RNAs.^21^, ^56^ Bai et al. proved that inhibition of YTHDF1 resulted in a remarkable decrease in Wnt/β‐catenin pathway activity in CRC cells.^57^ Additionally, the recognition of m^6^A methylation on YAP mRNA and its role in promoting translation have been linked to YTHDF1.^55^ Conversely, YTHDF2 typically accelerates mRNA decay.^21^, ^58^ Zhou et al. reported that YTHDF2 knockdown significantly elevated TRIM7 mRNA levels in osteosarcoma.^53^ Additionally, YTHDF2 recognises the m^6^A methylation of pre‐miR‐181b‐1, leading to RNA degradation.^55^ Similarly, our investigation validated YTHDF1 as an ‘reader’ of PRKDC m^6^A methylation. We demonstrated that YTHDF1 knockdown decreased PRKDC mRNA and DNA‐PKcs protein levels and reversed the changes in osteosarcoma sensitivity to anlotinib induced by METTL3 overexpression. Our YTHDF1 RIP assay revealed direct binding of YTHDF1 to PRKDC mRNA, regulated by METTL3. In addition, YTHDF1 knockdown decreased METTL3 induced PRKDC m^6^A methylation. These findings suggest that YTHDF1 recognises PRKDC m^6^A modifications and exerts a positive regulatory effect on its expression, thereby sustaining anlotinib resistance in osteosarcoma, as facilitated by METTL3. Notably, in U‐2 OS cells, reduced levels of DNA‐PKcs were observed in the YTHDF2 knockdown group. Given its role in RNA degradation, we speculated that YTHDF2 might regulate DNA‐PKcs through an alternative mechanism that requires further research and exploration.

On the whole, this research revealed a new function for METTL3 in anlotinib resistance in osteosarcoma, demonstrating that METTL3 enhances DNA‐PKcs expression through m^6^A methylation of PRKDC mRNA. Additionally, we observed that DNA‐PKcs contribute to anlotinib resistance in osteosarcoma cells by interacting directly with Beclin‐1 and inhibiting its ubiquitination. However, this study has several limitations. First, we have not elucidated the mechanism by which DNA‐PKcs inhibit Beclin‐1 ubiquitination or the underlying mechanism of anlotinib‐induced autophagy. In addition, the function of METTL3 in regulating DNA‐PKcs remains unclear. Although METTL14 was not upregulated by anlotinib treatment, it may be involved in PRKDC m^6^A modifications. Moreover, the effects of DNA‐PKcs on the downstream effects of tyrosine kinase inhibitors require further investigation. These questions require further investigation. Furthermore, this study only assessed the sensibility of osteosarcoma cells to anlotinib after treatment. Future research should focus on developing anlotinib‐resistant osteosarcoma cell lines to gain deeper perceptions of the molecular mechanisms of resistance to drugs.

## 5 CONCLUSION

Our findings provide a new understanding of anlotinib resistance in osteosarcoma. Potential therapeutic opportunities may arise by focusing on networks involving METTL3 and DNA‐PKcs, which could enhance the effectiveness of anlotinib and combat drug resistance in patients with osteosarcoma. Further studies exploring the clinical implications of these discoveries and advancements in precise treatments are essential to enhance the outlook of patients with osteosarcoma.

## AUTHOR CONTRIBUTIONS

Yining Zhang: Conceptualisation, Investigation, Methodology, Writing—original draft. Guohong Shen, Dan Zhang and Tingting Meng: Formal analysis. Zhaorui Lv and Lei Chen: Data curation. Jianmin Li: Supervision. Ka Li: Funding acquisition, Writing—review & editing and Project administration. All the authors have read and approved the final version of the manuscript.

## CONFLICT OF INTEREST STATEMENT

The authors declare that they have no conflict of interest.

## ETHICS STATEMENT

All animal experimental procedures and protocols were carried out according to the animal care standards of the Chinese National Institute of Health. These animal experiments were approved by the Research Ethics Committee of Shandong University Qilu Hospital (Jinan, China).

All human osteosarcoma tissues were collected from patients after surgical resection in Qilu Hospital of Shandong University. These patients did not undergo any anticancer treatment. All patients signed informed consent forms. The protocols were approved by the Research Ethics Committee of Shandong University Qilu Hospital.

## Supporting information



Supporting Information

## Data Availability

The data generated in this study are available upon request from the corresponding author.
