# Supplementary material for: N6‐Methyladenosine modification mediated by METTL3 promotes DNA‐PKcs expression to induce anlotinib resistance in osteosarcoma
Source: Clin Transl Med. 2025 Feb 9;15(2):e70228. doi: 10.1002/ctm2.70228 (PMC11807765; doi:10.1002/ctm2.70228)
Supplement: Supplementary file 1 — Supporting Information [file CTM2-15-e70228-s001.docx]

**Supplementary Table**

**Table S1** Plasmid sequence used in the study.

| Gene | Sequence (Vector: pcDNA3.4-3xflag-C) |
| --- | --- |
| METTL3 | atgtcggacacgtggagctctatccaggcccacaagaagcagctggactctctgcgggagaggctgcagcggaggcggaagcaggactcggggcacttggatctacggaatccagaggcagcattgtctccaaccttccgtagtgacagcccagtgcctactgcacccacctctggtggccctaagcccagcacagcttcagcagttcctgaattagctacagatcctgagttagagaagaagttgctacaccacctctctgatctggccttaacattgcccactgatgctgtgtccatctgtcttgccatctccacgccagatgctcctgccactcaagatggggtagaaagcctcctgcagaagtttgcagctcaggagttgattgaggtaaagcgaggtctcctacaagatgatgcacatcctactcttgtaacctatgctgaccattccaagctctctgccatgatgggtgctgtggcagaaaagaagggccctggggaggtagcagggactgtcacagggcagaagcggcgtgcagaacaggactcgactacagtagctgcctttgccagttcgttagtctctggtctgaactcttcagcatcggaaccagcaaaggagccagccaagaaatcaaggaaacatgctgcctcagatgttgatctggagatagagagccttctgaaccaacagtccactaaggaacaacagagcaagaaggtcagtcaggagatcctagagctattaaatactacaacagccaaggaacaatccattgttgaaaaatttcgctctcgaggtcgggcccaagtgcaagaattctgtgactatggaaccaaggaggagtgcatgaaagccagtgatgctgatcgaccctgtcgcaagctgcacttcagacgaattatcaataaacacactgatgagtctttaggtgactgctctttccttaatacatgtttccacatggatacctgcaagtatgttcactatgaaattgatgcttgcatggattctgaggcccctggcagcaaagaccacacgccaagccaggagcttgctcttacacagagtgtcggaggtgattccagtgcagaccgactcttcccacctcagtggatctgttgtgatatccgctacctggacgtcagtatcttgggcaagtttgcagttgtgatggctgacccaccctgggatattcacatggaactgccctatgggaccctgacagatgatgagatgcgcaggctcaacatacccgtactacaggatgatggctttctcttcctctgggtcacaggcagggccatggagttggggagagaatgtctaaacctctgggggtatgaacgggtagatgaaattatttgggtgaagacaaatcaactgcaacgcatcattcggacaggccgtacaggtcactggttgaaccatgggaaggaacactgcttggttggtgtcaaaggaaatccccaaggcttcaaccagggtctggattgtgatgtgatcgtagctgaggttcgttccaccagtcataaaccagatgaaatctatggcatgattgaaagactatctcctggcactcgcaagattgagttatttggacgaccacacaatgtgcaacccaactggatcacccttggaaaccaactggatgggatccacctactagacccagatgtggttgcacggttcaagcaaaggtacccagatggtatcatctctaaacctaagaattta |

**Table S2** siRNAs sequence used in the study.

| siRNA | Sequence |
| --- | --- |
| st-h-PRKDC | GCATCAGGGTTTAATCAGA |
| st-h-METTL3_001 | CAAGTATGTTCACTATGAA |
| st-h-METTL3_002 | GACTGCTCTTTCCTTAATA |
| st-h-METTL3_003 | GGACTCGACTACAGTAGCT |
| st-h-YTHDF1_001 | ACGGCAGAGTCGAAACAAA |
| st-h-YTHDF1_002 | CTCCACCCATAAAGCATAA |
| st-h-YTHDF1_003 | GCCGTCCATTGGATTTCCT |
| st-h-YTHDF2_001 | GACCAAGAATGGCATTGCA |
| st-h-YTHDF2_002 | GCACAGAAGTTGCAAGCAA |
| st-h-YTHDF2_003 | GGTAGCGGGTCCATTACTA |

**Table S3** Lentiviru Vectors sequence used in the study.

| Lentiviru | Sequence |
| --- | --- |
| LV-METTL3  (Ubi-MCS-3FLAG-SV40-Cherry-IRES-neomycin) | TTCTGGCCGTTTTTGGCTTTTTTGTTAGACGAAGCTTGGGCTGCAGGTCGACTCTAGAGGATCCCGCCACCATGTCGGACACGTGGAGCTCTATCCAGGCCCACAAGAAGCAGCTGGACTCTCTGCGGGAGAGGCTGCAGCGGAGGCGGAAGCAGGACTCGGGGCACTTGGATCTACGGAATCCAGAGGCAGCATTGTCTCCAACCTTCCGTAGTGACAGCCCAGTGCCTACTGCACCCACCTCTGGTGGCCCTAAGCCCAGCACAGCTTCAGCAGTTCCTGAATTAGCTACAGATCCTGAGTTAGAGAAGAAGTTGCTACACCACCTCTCTGATCTGGCCTTAACATTGCCCACTGATGCTGTGTCCATCTGTCTTGCCATCTCCACGCCAGATGCTCCTGCCACTCAAGATGGGGTAGAAAGCCTCCTGCAGAAGTTTGCAGCTCAGGAGTTGATTGAGGTAAAGCGAGGTCTCCTACAAGATGATGCACATCCTACTCTTGTAACCTATGCTGACCATTCCAAGCTCTCTGCCATGATGGGTGCTGTGGCAGAAAAGAAGGGCCCTGGGGAGGTAGCAGGGACTGTCACAGGGCAGAAGCGGCGTGCAGAACAGGACTCGACTACAGTAGCTGCCTTTGCCAGTTCGTTAGTCTCTGGTCTGAACTCTTCAGCATCGGAACCAGCAAAGGAGCCAGCCAAGAAATCAAGGAAACATGCTGCCTCAGATGTTGATCTGGAGATAGAGAGCCTTCTGAACCAACAGTCCACTAAGGAACAACAGAGCAAGAAGGTCAGTCAGGAGATCCTAGAGCTATTAAATACTACAACAGCCAAGGAACAATCCATTGTTGAAAAATTTCGCTCTCGAGGTCGGGCCCAAGTGCAAGAATTCTGTGACTATGGAACCAAGGAGGAGTGCATGAAAGCCAGTGATGCTGATCGACCCTGTCGCAAGCTGCACTTCAGACGAATTATCAATAAACACACTGATGAGTCTTTAGGTGACTGCTCTTTCCTTAATACATGTTTCCACATGGATACCTGCAAGTATGTTCACTATGAAATTGATGCTTGCATGGATTCTGAGGCCCCTGGCAGCAAAGACCACACGCCAAGCCAGGAGCTTGCTCTTACACAGAGTGTCGGAGGTGATTCCAGTGCAGACCGACTCTTCCCACCTCAGTGGATCTGTTGTGATATCCGCTACCTGGACGTCAGTATCTTGGGCAAGTTTGCAGTTGTGATGGCTGACCCACCCTGGGATATTCACATGGAACTGCCCTATGGGACCCTGACAGATGATGAGATGCGCAGGCTCAACATACCCGTACTACAGGATGATGGCTTTCTCTTCCTCTGGGTCACAGGCAGGGCCATGGAGTTGGGGAGAGAATGTCTAAACCTCTGGGGGTATGAACGGGTAGATGAAATTATTTGGGTGAAGACAAATCAACTGCAACGCATCATTCGGACAGGCCGTACAGGTCACTGGTTGAACCATGGGAAGGAACACTGCTTGGTTGGTGTCAAAGGAAATCCCCAAGGCTTCAACCAGGGTCTGGATTGTGATGTGATCGTAGCTGAGGTTCGTTCCACCAGTCATAAACCAGATGAAATCTATGGCATGATTGAAAGACTATCTCCTGGCACTCGCAAGATTGAGTTATTTGGACGACCACACAATGTGCAACCCAACTGGATCACCCTTGGAAACCAACTGGATGGGATCCACCTACTAGACCCAGATGTGGTTGCACGGTTCAAGCAAAGGTACCCAGATGGTATCATCTCTAAACCTAAGAATTTAACCGGTATGGACTACAAGGATGACGATGACAAGGATTACAAAGACGACGATGATAAGGACTATAAGGATGATGACGACAAATGAGCTAGCCTGTGGAATGTGTGTCAGTTAGGGTGTGGAAAGTCCCCAGGCTCCCCAGCAGGCAGAAGTATGCAAAGCATGCATCTCAATTA |
| LV-shPRKDC  (hU6-MCS-CBh-gcGFP-IRES-puromycin) | ccggccGGTAAAGATCCTAATTCTActcgagTAGAATTAGGATCTTTACCggtttttg |
| LV-RFP-GFP-LC3  (hU6-MCS-Ubiquitin-stubRFP-senseGFP-LC3-IRES-puromycin) | atggtgtctaagggcgaagagctgattaaggagaacatgcacatgaagctgtacatggagggcaccgtgaacaaccaccacttcaagtgcacatccgagggcgaaggcaagccctacgagggcacccagaccatgagaatcaaggtggtcgagggcggccctctccccttcgccttcgacatcctggctaccagcttcatgtacggcagcagaaccttcatcaaccacacccagggcatccccgacttctttaagcagtccttccctgagggcttcacatgggagagagtcaccacatacgaagacgggggcgtgctgaccgctacccaggacaccagcctccaggacggctgcctcatctacaacgtcaagatcagaggggtgaacttcccatccaacggccctgtgatgcagaagaaaacactcggctgggaggccaacaccgagatgctgtaccccgctgacggcggcctggaaggcagaagcgacatggccctgaagctcgtgggcgggggccacctgatctgcaacttcaagaccacatacagatccaagaaacccgctaagaacctcaagatgcccggcgtctactatgtggaccacagactggaaagaatcaaggaggccgacaaagagacctacgtcgagcagcacgaggtggctgtggccagatactgcgacctccctagcaaactggggcacaaacttaatgcggccgctatggtgagcaagggcgaggagaccacaatgggcgtaatcaagcccgacatgaagatcaagctgaagatggagggcaacgtgaatggccacgccttcgtgatcgagggcgagggcgagggcaagccctacgacggcaccaacaccatcaacctggaggtgaaggagggagcccccctgcccttctcctacgacattctgaccaccgcgttcagttacggcaacagggccttcaccaagtaccccgacgacatccccaactacttcaagcagtccttccccgagggctactcttgggagcgcaccatgaccttcgaggacaagggcatcgtgaaggtgaagtccgacatctccatggaggaggactccttcatctacgagatacacctcaagggcgagaacttcccccccaacggccccgtgatgcagaaggagaccaccggctgggacgcctccaccgagaggatgtacgtgcgcgacggcgtgctgaagggcgacgtcaagatgaagctgctgctggagggcggcggccaccaccgcgttgacttcaagaccatctacagggccaagaaggcggtgaagctgcccgactatcactttgtggaccaccgcatcgagatcctgaaccacgacaaggactacaacaaggtgaccgtttacgagatcgccgtggcccgcaactccaccgacggcatggacgagctgtacaagatcgattccggaatgccgtcggagaagaccttcaagcagcgccgcaccttcgaacaaagagtagaagatgtccgacttattcgagagcagcatccaaccaaaatcccggtgataatagaacgatacaagggtgagaagcagcttcctgttctggataaaacaaagttccttgtacctgaccatgtcaacatgagtgagctcatcaagataattagaaggcgcttacagctcaatgctaatcaggccttcttcctgttggtgaacggacacagcatggtcagcgtctccacaccaatctcagaggtgtatgagagtgagaaagatgaagatggattcctgtacatggtctatgcctcccaggagacgttcgggatgaaattgtcagtgtaa |

**Table S4** Probe sequence used for FISH.

| Gene | Sequence |
| --- | --- |
| PRKDC | CTGGAGGTCATGGGCACAACGCTATAGGTCCTCAGCTGCAGGGCCCTCTGGCTGCAGGCGGAGTCTTGGGCCAGGATCCCATTCATGACCTGGAAGAGCTGCTCCACGCGCTGGTCCTGCCGCAGGTCCTCGCCACCCTTCACCAGGAAAGGGTGTTCCCTCTCGTCATGGCCACGGATGATGATGCGCTTGGGCCTTCGCAGAGACGCCATGACTGTCAC |

**Table S5** Plasmid sequence used for Dual-luciferase reporter.

|  | Plasmid for Dual-luciferase reporter; Vector: pmirGLO |
| --- | --- |
| PRKDC P1(WT) | GACGCCCGCAAGATCCGCGAGATTCTCATTAAGGCCAAGAAGGGCGGCAAGATCGCCGTGTAATTCTAGTTGTTTAAACGAGCTCATTCGAACAACAAGGAGTTATCTATTGCTATCCGTGGATATGGACTTTTTGCAGGACCGTGCAAGGTTATAAACGCAAAAGATGTTGCTCGAGTCTAGAGTCGACCTGCAGGCATGCAAGCTGATCCGGCTGCTAACAAAGCCCGAAAGGAAGCTGAGTTGGCTGCTGCCACCGCTGAGCAATAACTAGCATAACCCCTTGGGGCGGCCGCTTCGAGCAGACATGATAAGATACATTGATGAGTTTGGACAAACCACAACTAGAATGCAGTGAAAAAAATGCTTTATTTGTGAAATTTGTGATGCTATTGCTTTATTTGTAACCATTATAAGCTGCAATAAACAAGTTAACAACAACAATTGCATTCATTTTATGTTTCAGGTTCAGGGGGAGATGTGGGAGGTTTTTTTAAGCAAGTAAAACCTCTACAAATGTGGTAAAATCGAATTTTAACAAAATATTAACGCTTACAATTTCCTGATGCGGTATTTTCTCCTTACGCATCTGTGCGGTATTTCACACCGCATACGCGGATCTGCGCAGCACCATGGCCTGAAATAACCTCTGAAAGAGGAACTTGGTTAGGTACCTTCTGAGGCGGAAAGAACCAGCTGTGGAATGTGTGTCAGTTAGGGTGTGGAAAGTCCCCAGGCTCCCCAGCAGGCAGAAGTATGCAaAGCATGCATCTCAATTAGTCAGCAAC |
| PRKDC P1(MUT) | GCCCGCAAGATCCGCGAGATTCTCATTAAGGCCAAGAAGGGCGGCAAGATCGCCGTGTAATTCTAGTTGTTTAAACGAGCTCATTCGAACAACAAGGAGTTATCTATTGCTATCCGTGGATATGGTCTTTTTGCAGGACCGTGCAAGGTTATAAACGCAAAAGATGTTGCTCGAGTCTAGAGTCGACCTGCAGGCATGCAAGCTGATCCGGCTGCTAACAAAGCCCGAAAGGAAGCTGAGTTGGCTGCTGCCACCGCTGAGCAATAACTAGCATAACCCCTTGGGGCGGCCGCTTCGAGCAGACATGATAAGATACATTGATGAGTTTGGACAAACCACAACTAGAATGCAGTGAAAAAAATGCTTTATTTGTGAAATTTGTGATGCTATTGCTTTATTTGTAACCATTATAAGCTGCAATAAACAAGTTAACAACAACAATTGCATTCATTTTATGTTTCAGGTTCAGGGGGAGATGTGGGAGGTTTTTTTAAGCAAGTAAAACCTCTACAAATGTGGTAAAATCGAATTTTAACAAAATATTAACGCTTACAATTTCCTGATGCGGTATTTTCTCCTTACGCATCTGTGCGGTATTTCACACCGCATACGCGGATCTGCGCAGCACCATGGCCTGAAATAACCTCTGAAAGAGGAACTTGGTTAGGTACCTTCTGAGGCGGAAAGAACCAGCTGTGGAATGTGTGTCAGTTAGGGTGTGGAAAGTCCCCAGGCTCCCCAGCAGGCAGAAGTATGCAAAGCATGCATCTCAATTAGTCAGCAACCAG |
| PRKDC P2(WT) | TGGACGCCCGCAAGATCCGCGAGATTCTCATTAAGGCCAAGAAGGGCGGCAAGATCGCCGTGTAATTCTAGTTGTTTAAACGAGCTCCACGACTCTGCTAAACACCTCCCCGGAAGGATGGAAGCTCCTGAAGAAGGACTTGTGTAATACACACCTGATGAGAGTCCTGGTGCAGACGCTGTGTGAGCCTCGAGTCTAGAGTCGACCTGCAGGCATGCAAGCTGATCCGGCTGCTAACAAAGCCCGAAAGGAAGCTGAGTTGGCTGCTGCCACCGCTGAGCAATAACTAGCATAACCCCTTGGGGCGGCCGCTTCGAGCAGACATGATAAGATACATTGATGAGTTTGGACAAACCACAACTAGAATGCAGTGAAAAAAATGCTTTATTTGTGAAATTTGTGATGCTATTGCTTTATTTGTAACCATTATAAGCTGCAATAAACAAGTTAACAACAACAATTGCATTCATTTTATGTTTCAGGTTCAGGGGGAGATGTGGGAGGTTTTTTTAAGCAAGTAAAACCTCTACAAATGTGGTAAAATCGAATTTTAACAAAATATTAACGCTTACAATTTCCTGATGCGGTATTTTCTCCTTACGCATCTGTGCGGTATTTCACACCGCATACGCGGATCTGCGCAGCACCATGGCCTGAAATAACCTCTGAAAGAGGAACTTGGTTAGGTACCTTCTGAGGCGGAAAGAACCAGCTGTGGAATGTGTGTCAGTTAGGGTGTGGAAAGTCCCCAGGCTCCCCAGCAGGCAGAAGTATGCAAAGCATGCATC |
| PRKDC P2(MUT) | GGACGCCCGCAAGATCCGCGAGATTCTCATTAAGGCCAAGAAGGGCGGCAAGATCGCCGTGTAATTCTAGTTGTTTAAACGAGCTCCACGACTCTGCTAAACACCTCCCCGGAAGGATGGAAGCTCCTGAAGAAGGTCTTGTGTAATACACACCTGATGAGAGTCCTGGTGCAGACGCTGTGTGAGCCTCGAGTCTAGAGTCGACCTGCAGGCATGCAAGCTGATCCGGCTGCTAACAAAGCCCGAAAGGAAGCTGAGTTGGCTGCTGCCACCGCTGAGCAATAACTAGCATAACCCCTTGGGGCGGCCGCTTCGAGCAGACATGATAAGATACATTGATGAGTTTGGACAAACCACAACTAGAATGCAGTGAAAAAAATGCTTTATTTGTGAAATTTGTGATGCTATTGCTTTATTTGTAACCATTATAAGCTGCAATAAACAAGTTAACAACAACAATTGCATTCATTTTATGTTTCAGGTTCAGGGGGAGATGTGGGAGGTTTTTTTAAGCAAGTAAAACCTCTACAAATGTGGTAAAATCGAATTTTAACAAAATATTAACGCTTACAATTTCCTGATGCGGTATTTTCTCCTTACGCATCTGTGCGGTATTTCACACCGCATACGCGGATCTGCGCAGCACCATGGCCTGAAATAACCTCTGAAAGAGGAACTTGGTTAGGTACCTTCTGAGGCGGAAAGAACCAGCTGTGGAATGTGTGTCAGTTAGGGTGTGGAAAGTCCCCAGGCTCCCCAGCAGGCAGAAGTATGCAAAGCATGCATCTCAAT |
| PRKDC P3(WT) | GGACGCCCGCAAGATCCGCGAGATTCTCATTAAGGCCAAGAAGGGCGGCAAGATCGCCGTGTAATTCTAGTTGTTTAAACGAGCTCTCCTTTGTAAGATATAAAGAAGTGTATGCCGCTGCAGCAGAAGTTCTAGGACTTATACTTCGATATGTTATGGAGAGAAAAAACATACTGGAGGAGTCTCTCTCGAGTCTAGAGTCGACCTGCAGGCATGCAAGCTGATCCGGCTGCTAACAAAGCCCGAAAGGAAGCTGAGTTGGCTGCTGCCACCGCTGAGCAATAACTAGCATAACCCCTTGGGGCGGCCGCTTCGAGCAGACATGATAAGATACATTGATGAGTTTGGACAAACCACAACTAGAATGCAGTGAAAAAAATGCTTTATTTGTGAAATTTGTGATGCTATTGCTTTATTTGTAACCATTATAAGCTGCAATAAACAAGTTAACAACAACAATTGCATTCATTTTATGTTTCAGGTTCAGGGGGAGATGTGGGAGGTTTTTTTAAGCAAGTAAAACCTCTACAAATGTGGTAAAATCGAATTTTAACAAAATATTAACGCTTACAATTTCCTGATGCGGTATTTTCTCCTTACGCATCTGTGCGGTATTTCACACCGCATACGCGGATCTGCGCAGCACCATGGCCTGAAATAACCTCTGAAAGAGGAACTTGGTTAGGTACCTTCTGAGGCGGAAAGAACCAGCTGTGGAATGTGTGTCAGTTAGGGTGTGGAAAGTCCCCAGGCTCCCCAGCAGGCAGAAGTATGCAAAGCATGCATCTC |
| PRKDC P3(MUT) | TGGACGCCCGCAAGATCCGCGAGATTCTCATTAAGGCCAAGAAGGGCGGCAAGATCGCCGTGTAATTCTAGTTGTTTAAACGAGCTCTCCTTTGTAAGATATAAAGAAGTGTATGCCGCTGCAGCAGAAGTTCTAGGTCTTATACTTCGATATGTTATGGAGAGAAAAAACATACTGGAGGAGTCTCTCTCGAGTCTAGAGTCGACCTGCAGGCATGCAAGCTGATCCGGCTGCTAACAAAGCCCGAAAGGAAGCTGAGTTGGCTGCTGCCACCGCTGAGCAATAACTAGCATAACCCCTTGGGGCGGCCGCTTCGAGCAGACATGATAAGATACATTGATGAGTTTGGACAAACCACAACTAGAATGCAGTGAAAAAAATGCTTTATTTGTGAAATTTGTGATGCTATTGCTTTATTTGTAACCATTATAAGCTGCAATAAACAAGTTAACAACAACAATTGCATTCATTTTATGTTTCAGGTTCAGGGGGAGATGTGGGAGGTTTTTTTAAGCAAGTAAAACCTCTACAAATGTGGTAAAATCGAATTTTAACAAAATATTAACGCTTACAATTTCCTGATGCGGTATTTTCTCCTTACGCATCTGTGCGGTATTTCACACCGCATACGCGGATCTGCGCAGCACCATGGCCTGAAATAACCTCTGAAAGAGGAACTTGGTTAGGTACCTTCTGAGGCGGAAAGAACCAGCTGTGGAATGTGTGTCAGTTAGGGTGTGGAAAGTCCCCAGGCTCCCCAGCAGGCAGAAGTATGCAAAGCATGCATCT |
| PRKDC P4(WT) | GCCCGCAAGATCCGCGAGATTCTCATTAAGGCCAAGAAGGGCGGCAAGATCGCCGTGTAATTCTAGTTGTTTAAACGAGCTCCTTCAACGACATTACCAACATGCTACTTTTAAAAATGAACAAAGACTCAAAGCCCCCTGGGAATCTGAAAGAATGTTCACCCTGGATGACTCGAGTCTAGAGTCGACCTGCAGGCATGCAAGCTGATCCGGCTGCTAACAAAGCCCGAAAGGAAGCTGAGTTGGCTGCTGCCACCGCTGAGCAATAACTAGCATAACCCCTTGGGGCGGCCGCTTCGAGCAGACATGATAAGATACATTGATGAGTTTGGACAAACCACAACTAGAATGCAGTGAAAAAAATGCTTTATTTGTGAAATTTGTGATGCTATTGCTTTATTTGTAACCATTATAAGCTGCAATAAACAAGTTAACAACAACAATTGCATTCATTTTATGTTTCAGGTTCAGGGGGAGATGTGGGAGGTTTTTTTAAGCAAGTAAAACCTCTACAAATGTGGTAAAATCGAATTTTAACAAAATATTAACGCTTACAATTTCCTGATGCGGTATTTTCTCCTTACGCATCTGTGCGGTATTTCACACCGCATACGCGGATCTGCGCAGCACCATGGCCTGAAATAACCTCTGAAAGAGGAACTTGGTTAGGTACCTTCTGAGGCGGAAAGAACCAGCTGTGGAATGTGTGTCAGTTAGGGTGTGGAAAGTCCCCAGGCTCCCCAGCAGGCAGAAGTATGCAAAGCATGCATCTCAATTAGTCAGCAACC |
| PRKDC P4(MUT) | CGCCCGCAAGATCCGCGAGATTCTCATTAAGGCCAAGAAGGGCGGCAAGATCGCCGTGTAATTCTAGTTGTTTAAACGAGCTCCTTCAACGACATTACCAACATGCTACTTTTAAAAATGAACAAAGTCTCAAAGCCCCCTGGGAATCTGAAAGAATGTTCACCCTGGATGACTCGAGTCTAGAGTCGACCTGCAGGCATGCAAGCTGATCCGGCTGCTAACAAAGCCCGAAAGGAAGCTGAGTTGGCTGCTGCCACCGCTGAGCAATAACTAGCATAACCCCTTGGGGCGGCCGCTTCGAGCAGACATGATAAGATACATTGATGAGTTTGGACAAACCACAACTAGAATGCAGTGAAAAAAATGCTTTATTTGTGAAATTTGTGATGCTATTGCTTTATTTGTAACCATTATAAGCTGCAATAAACAAGTTAACAACAACAATTGCATTCATTTTATGTTTCAGGTTCAGGGGGAGATGTGGGAGGTTTTTTTAAGCAAGTAAAACCTCTACAAATGTGGTAAAATCGAATTTTAACAAAATATTAACGCTTACAATTTCCTGATGCGGTATTTTCTCCTTACGCATCTGTGCGGTATTTCACACCGCATACGCGGATCTGCGCAGCACCATGGCCTGAAATAACCTCTGAAAGAGGAACTTGGTTAGGTACCTTCTGAGGCGGAAAGAACCAGCTGTGGAATGTGTGTCAGTTAGGGTGTGGAAAGTCCCCAGGCTCCCCAGCAGGCAGAAGTATGCAAAGCATGCATCTCAATTAGTCAGCAAC |

**Supplementary Figure**


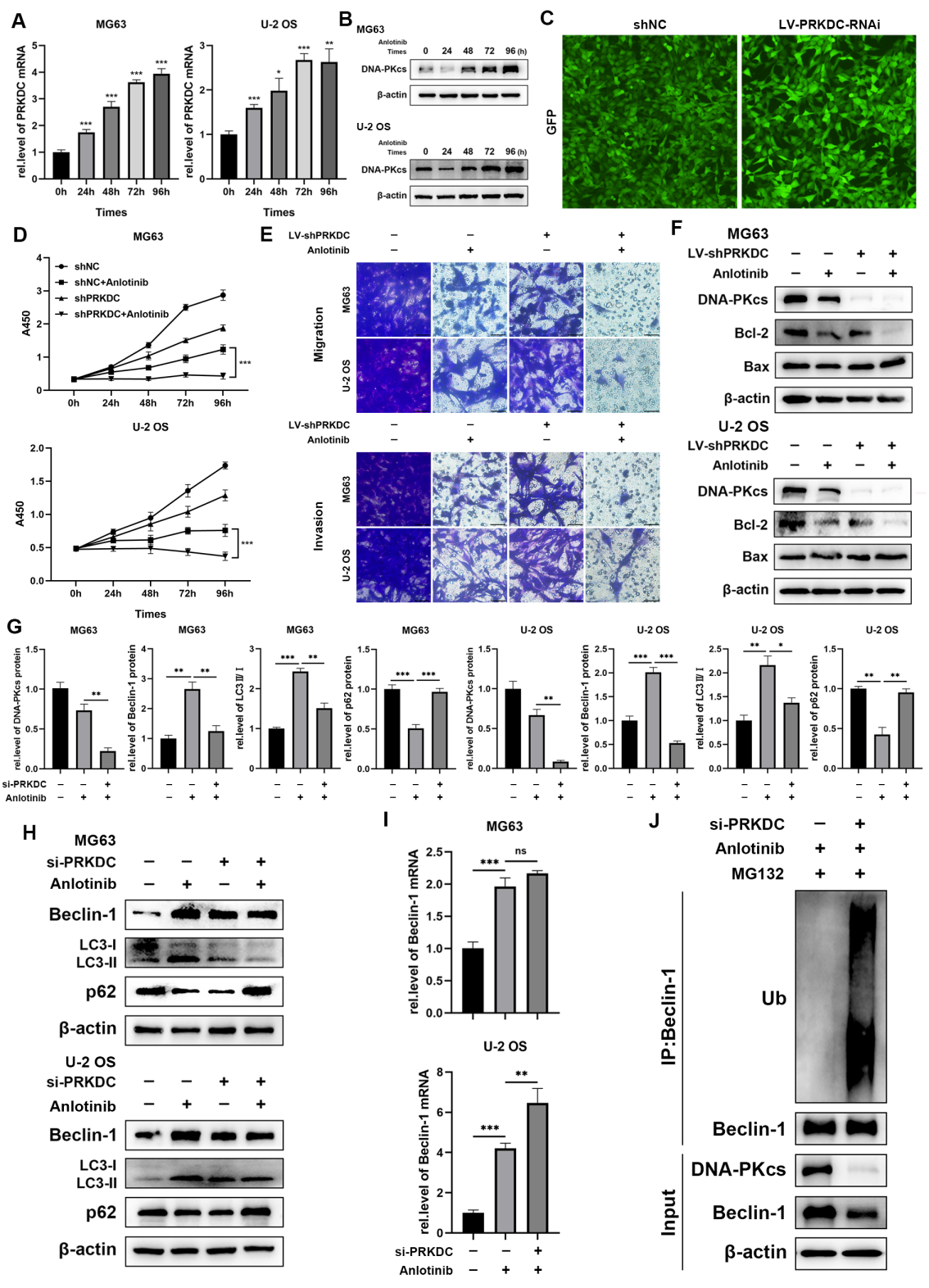


**Figure S1** **DNA-PKcs was related with osteosarcoma cell response to anlotinib.** (A) The qRT-PCR assay measured PRKDC mRNA expression in MG63 and U-2 OS cells following anlotinib treatment for 24, 48, 72, and 96 hours.. (B) Western blot analysis showed DNA-PKcs protein expression in MG63 and U-2 OS cells after incubation with anlotinib for 24, 48, 72, and 96 hours. (C) Representative images of cells (green) detected by fluorescence microscope, Scale bar = 200μm. MG63 and U-2 OS cells were stably transfected with PRKDC shRNA or a control shRNA lentivirus before being treated with anlotinib. (D) The CCK8 assay assessed the viability of MG63 and U-2 OS cells after the indicated treatments. (E) Transwell assay, without or with Matrigel, determined the migration and invasion capabilities of the cells, scale bar = 50μm. (F) Western blotting assessed protein levels of DNA-PKcs, Bcl-2, Bax in MG63 and U-2 OS cells. MG63 and U-2 OS cells were transfected with PRKDC siRNA and subsequently treated with anlotinib. (G) Quantification of DNA PKcs, Beclin-1, LC3 and p62 protein expression in MG63 and U-2 OS cells. (H) Western blot analysis was used to detect the expression of autophagy-related proteins in MG63 and U-2 OS cells. (I) qRT-PCR was used to determine the Beclin-1 mRNA level in MG63 and U-2 OS cells after treated with si-NC or si-PRKDC and anlotinib. (J) Beclin-1 IP and western blot analysis assessed Beclin-1 ubiquitination in MG63 cells following DNA-PKcs knockdown and MG132 treatment. The data are expressed as mean ± SD, n = 3. **P < 0.01; ***P < 0.001 (one-way ANOVA followed by Bonferroni post-tests).


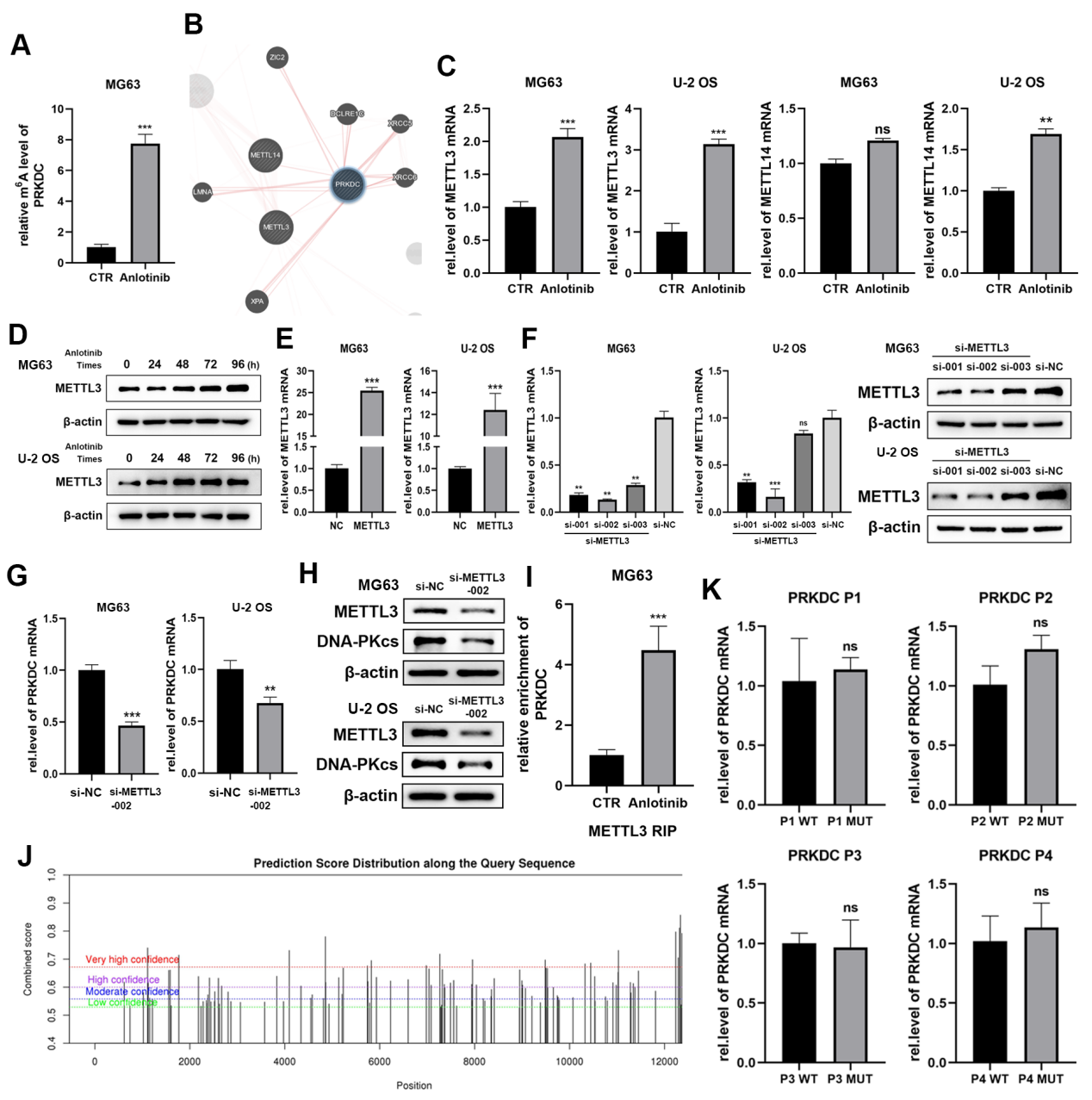


**Figure S2 METTL3 enhanced anlotinib resistance by mediating PRKDC m^6^A modification.** (A) MG63 cells were treated with Anlotinib, the m^6^A modification of PRKDC was determined using MeRIP-qPCR. (B) Bioinformatics analysis predicts an interaction between PRKDC and METTL3 or METTL14 (GeneMANIA). (C) The qRT-PCR assay detected METTL3 and METTL14 mRNA expression in MG63 and U-2 OS cells after anlotinib treatment. (D) Western blot analysis measured METTL3 protein levels in MG63 and U-2 OS cells following anlotinib treatment for 24, 48, 72, and 96 h. (E) METTL3 plasmid was transfected into MG63 and U-2 OS cells, qRT-PCR was used to detect the METTL3 mRNA expression. (F) Three types of METTL3 siRNA were transfected into cells. qRT-PCR was used to detect the METTL3 mRNA expression, and western blotting was used to detect the METTL3 protein expression. (G) The qRT-PCR assay assessed PRKDC mRNA expression in MG63 and U-2 OS cells transfected with METTL3 siRNA-002. (H) Western blot analysis measured METTL3 and DNA-PKcs protein levels in MG63 and U-2 OS cells following transfection with METTL3 siRNA-002. (I) MG63 cells were treated with Anlotinib, the enrichment of PRKDC was determined using METTL3 RIP. (J) The potential m^6^A positions of PRKDC mRNA predicted by SRAMP database (http://www.cuilab.cn/sramp). (K) Wild-type (WT) or mutant (MUT) double-luciferase reporter plasmids were transfected into MG63 cells, the qRT-PCR assay detected PRKdC mRNA expression. The data are expressed as mean ± SD, n = 3. **P < 0.01; ***P < 0.001 (Student’s t-test for two groups, one-way ANOVA followed by Bonferroni post-tests for multiply groups).


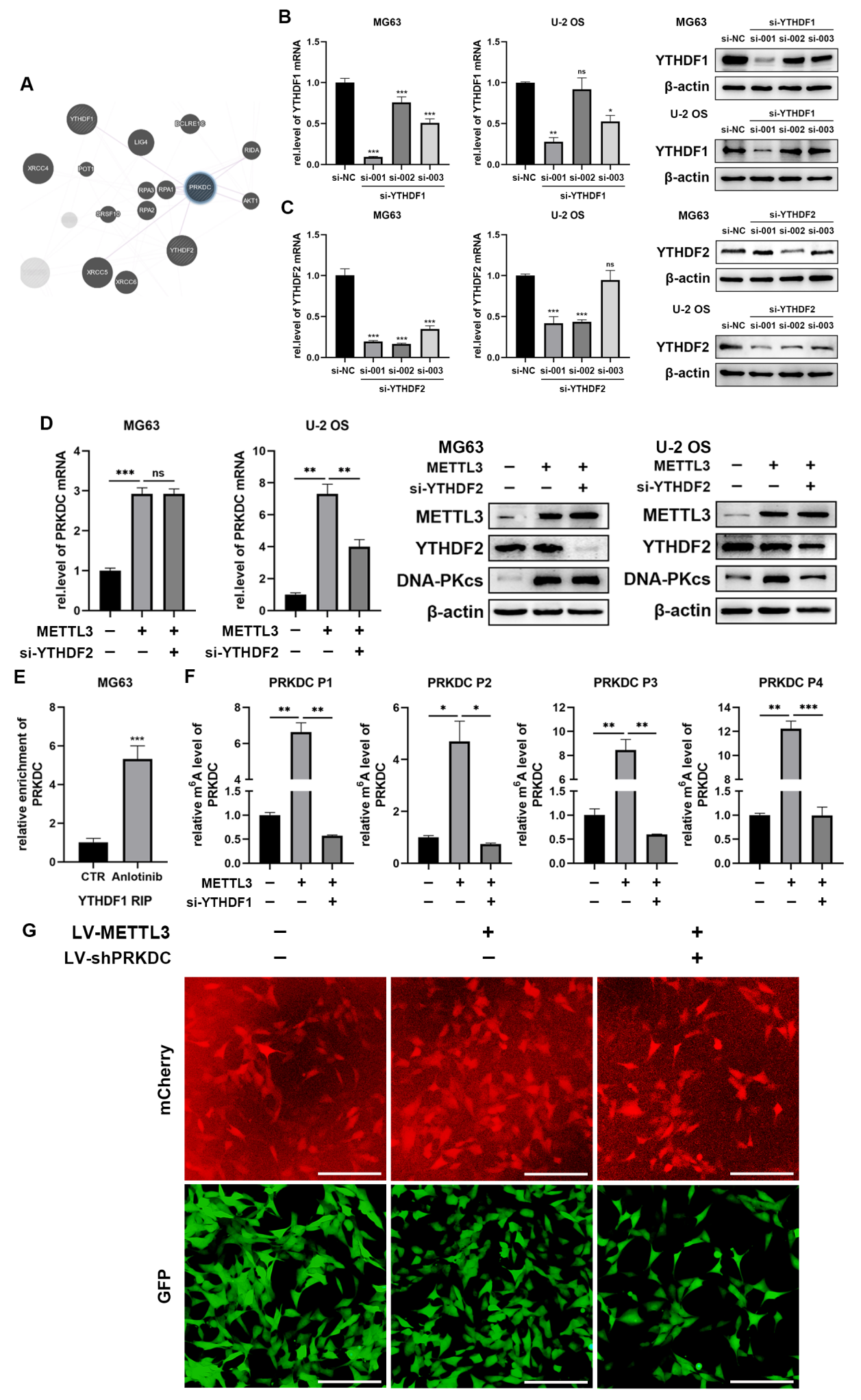


**Figure S3 YTHDF1 recognizes PRKDC m^6^A modification.** (A) Bioinformatics analysis predicts an interaction between PRKDC and YTHDF1 or YTHDF2 (GeneMANIA). (B) Three types of YTHDF1 siRNA were transfected into cells. The YTHDF1 mRNA level was detected using qRT-PCR, the YTHDF1 protein expression was detected using western blotting assay. (C) Three types of YTHDF2 siRNA were transfected into cells. qRT-PCR was used to detect the YTHDF2 mRNA expression, and western blotting was used to detect the YTHDF2 protein expression. Then chose the siRNA containing the highest transfection efficiency. (D) The qRT-PCR assay determined PRKDC mRNA expression in MG63 and U-2 OS cells co-transfected with METTL3 plasmid and YTHDF2 siRNA, western blotting analyzed METTL3, YTHDF2, and DNA-PKcs protein levels in MG63 and U-2 OS cells with METTL3 overexpression and YTHDF2 knockdown. (E) MG63 cells were treated with Anlotinib, the enrichment of PRKDC was determined using YTHDF1 RIP. (F) MG63 cells were co-transfected with the METTL3 plasmid and YTHDF1 siRNA or NC siRNA, then MeRIP-qPCR were used to measure the m^6^A modification levels in four specific PRKDC regions. (G) Representative images of cells (red: METTL3 or control lentivirus; green: PRKDC shRNA or control shRNA lentivirus) detected by fluorescence microscope, Scale bar = 200μm. The data are expressed as mean ± SD, n = 3. *P < 0.05; **P < 0.01; ***P < 0.001 (one-way ANOVA followed by Bonferroni post-tests).

**Bioinformatics predicts interaction between DNA-PKcs and Beclin-1 in HitPredict database**

*************Generated from HitPredict database (18Jul2023)*************

List of interactions for P78527|PRKDC|Homo sapiens|Interactions:579

************************************************************************

Interaction Interactor Name Experiments Category Method Score Annotation Score Interaction Score Confidence

490552 P13010 XRCC5 22 Small-scale 0.98 1.00 0.988 High

489384 P12956 XRCC6 16 Small-scale 0.96 1.00 0.982 High

864414 Q13418 ILK 3 High-throughput 0.76 1.00 0.871 High

864373 P78527 PRKDC 3 Small-scale 0.71 1.00 0.845 High

864566 Q99459 CDC5L 2 Small-scale 0.66 1.00 0.811 High

364296 P04637 P53 11 Small-scale 0.95 0.60 0.754 High

864562 Q96SD1 DCR1C 7 Small-scale 0.93 0.60 0.749 High

514463 P15927 RFA2 7 Small-scale 0.93 0.60 0.748 High

515924 P16104 H2AX 7 Small-scale 0.93 0.60 0.745 High

172673 O15524 SOCS1 2 High-throughput 0.54 1.00 0.737 High

549320 P20339 RAB5A 2 High-throughput 0.54 1.00 0.737 High

824081 P61586 RHOA 2 High-throughput 0.54 1.00 0.737 High

864413 Q13315 ATM 2 Small-scale 0.53 1.00 0.727 High

734915 P46531 NOTC1 1 High-throughput 0.51 1.00 0.715 High

128614 O00743 PPP6 4 Small-scale 0.82 0.60 0.703 High

601856 P27694 RFA1 4 Small-scale 0.80 0.60 0.694 High

855067 P68431 H31 4 Small-scale 0.78 0.60 0.683 High

242577 O75170 PP6R2 3 Small-scale 0.76 0.60 0.674 High

475097 P11387 TOP1 3 Small-scale 0.75 0.60 0.671 High

186462 O43156 TTI1 3 Small-scale 0.74 0.60 0.667 High

864422 Q13618 CUL3 3 High-throughput 0.74 0.60 0.667 High

864669 Q9Y4R8 TELO2 3 Small-scale 0.74 0.60 0.667 High

427284 P09874 PARP1 4 Small-scale 0.74 0.60 0.666 High

474071 P11308 ERG 2 Small-scale 0.73 0.60 0.664 High

864396 Q09472 EP300 3 Small-scale 0.73 0.60 0.660 High

864402 Q12905 ILF2 3 Small-scale 0.73 0.60 0.660 High

326906 P01106 MYC 3 High-throughput 0.72 0.60 0.656 High

556927 P21709 EPHA1 3 High-throughput 0.72 0.60 0.656 High

764672 P49917 DNLI4 3 Small-scale 0.71 0.60 0.655 High

494394 P13569 CFTR 3 High-throughput 0.70 0.60 0.650 High

864465 Q5S007 LRRK2 3 Small-scale 0.70 0.60 0.650 High

318623 P00519 ABL1 1 Small-scale 0.42 1.00 0.647 High

406844 P07948 LYN 1 Small-scale 0.42 1.00 0.647 High

626764 P31749 AKT1 2 Small-scale 0.70 0.60 0.646 High

864577 Q9BPZ7 SIN1 1 Small-scale 0.42 1.00 0.646 High

319453 P00533 EGFR 3 Small-scale 0.70 0.60 0.646 High

864619 Q9H9Q4 NHEJ1 3 Small-scale 0.68 0.60 0.641 High

214482 O60260 PRKN 3 High-throughput 0.68 0.60 0.640 High

456101 P10275 ANDR 3 Small-scale 0.68 0.60 0.640 High

864452 Q16665 HIF1A 3 Small-scale 0.68 0.60 0.640 High

864621 Q9HB75 PIDD1 2 Small-scale 0.65 0.60 0.624 High

864675 Q9Y6K9 NEMO 2 Small-scale 0.65 0.60 0.624 High

864415 Q13426 XRCC4 3 Small-scale 0.70 0.55 0.619 High

115310 O00221 IKBE 1 High-throughput 0.38 1.00 0.618 High

158496 O15084 ANR28 1 High-throughput 0.38 1.00 0.618 High

252811 O75530 EED 1 High-throughput 0.38 1.00 0.618 High

612436 P29353 SHC1 1 High-throughput 0.38 1.00 0.618 High

817980 P60953 CDC42 1 High-throughput 0.38 1.00 0.618 High

826678 P61964 WDR5 1 High-throughput 0.38 1.00 0.618 High

864378 Q00653 NFKB2 1 High-throughput 0.38 1.00 0.618 High

864525 Q8WWX0 ASB5 1 High-throughput 0.38 1.00 0.618 High

864541 Q969H0 FBXW7 1 High-throughput 0.38 1.00 0.618 High

864578 Q9BQ67 GRWD1 1 High-throughput 0.38 1.00 0.618 High

864608 Q9H4D1 Q9H4D1 1 High-throughput 0.38 1.00 0.618 High

864654 Q9UM11 FZR1 1 Small-scale 0.38 1.00 0.618 High

685361 P38398 BRCA1 2 Small-scale 0.63 0.60 0.613 High

864425 Q14191 WRN 2 Small-scale 0.63 0.60 0.613 High

469055 P11021 BIP 2 High-throughput 0.62 0.60 0.611 High

787781 P53350 PLK1 2 Small-scale 0.62 0.60 0.611 High

796614 P54646 AAPK2 2 Small-scale 0.62 0.60 0.611 High

864403 Q12906 ILF3 2 Small-scale 0.62 0.60 0.611 High

864553 Q96GD4 AURKB 2 High-throughput 0.62 0.60 0.611 High

864618 Q9H967 WDR76 2 High-throughput 0.62 0.60 0.611 High

313149 O96017 CHK2 2 Small-scale 0.60 0.60 0.602 High

504746 P14859 PO2F1 2 Small-scale 0.60 0.60 0.602 High

627130 P31751 AKT2 2 Small-scale 0.60 0.60 0.602 High

826119 P61956 SUMO2 2 High-throughput 0.60 0.60 0.602 High

567629 P23025 XPA 2 Small-scale 0.60 0.60 0.602 High

864494 Q86WJ1 CHD1L 2 Small-scale 0.60 0.60 0.601 High

864597 Q9GZX7 AICDA 2 Small-scale 0.65 0.55 0.598 High

598283 P27348 1433T 2 High-throughput 0.59 0.60 0.595 High

731811 P45984 MK09 2 Small-scale 0.59 0.60 0.595 High

348125 P03372 ESR1 2 Small-scale 0.59 0.60 0.594 High

362476 P04629 NTRK1 2 High-throughput 0.59 0.60 0.594 High

475557 P11388 TOP2A 2 Small-scale 0.58 0.60 0.588 High

425160 P09629 HXB7 1 Small-scale 0.57 0.60 0.586 High

864559 Q96RI1 NR1H4 1 Small-scale 0.57 0.60 0.586 High

864411 Q13286 CLN3 2 Small-scale 0.62 0.55 0.585 High

378368 P05412 JUN 2 Small-scale 0.57 0.60 0.585 High

481959 P11831 SRF 2 Small-scale 0.57 0.60 0.585 High

168736 O15392 BIRC5 2 Small-scale 0.57 0.60 0.584 High

230104 O60885 BRD4 2 High-throughput 0.57 0.60 0.584 High

287236 O95071 UBR5 2 Small-scale 0.57 0.60 0.584 High

470844 P11142 HSP7C 2 High-throughput 0.57 0.60 0.584 High

751178 P48729 KC1A 2 High-throughput 0.57 0.60 0.584 High

769341 P50750 CDK9 2 High-throughput 0.57 0.60 0.584 High

838793 P62805 H4 2 High-throughput 0.57 0.60 0.584 High

861054 P78317 RNF4 2 Small-scale 0.57 0.60 0.584 High

864387 Q04206 TF65 2 Small-scale 0.57 0.60 0.584 High

864409 Q13263 TIF1B 2 Small-scale 0.57 0.60 0.584 High

864508 Q8N6T7 SIR6 2 Small-scale 0.57 0.60 0.584 High

864588 Q9BXM7 PINK1 2 Small-scale 0.57 0.60 0.584 High

864661 Q9Y230 RUVB2 2 Small-scale 0.57 0.60 0.584 High

864574 Q99828 CIB1 2 Small-scale 0.61 0.55 0.581 High

720026 P42574 CASP3 2 Small-scale 0.60 0.55 0.577 High

864386 Q03112 MECOM 2 High-throughput 0.60 0.55 0.576 High

544932 P19838 NFKB1 1 High-throughput 0.33 1.00 0.575 High

719351 P42345 MTOR 1 Small-scale 0.33 1.00 0.575 High

808199 P57078 RIPK4 1 High-throughput 0.33 1.00 0.575 High

845344 P62993 GRB2 1 High-throughput 0.33 1.00 0.575 High

146288 O14757 CHK1 2 Small-scale 0.54 0.60 0.571 High

374868 P05161 ISG15 2 Small-scale 0.54 0.60 0.571 High

441814 P0C0S5 H2AZ 2 Small-scale 0.54 0.60 0.571 High

541923 P19419 ELK1 2 High-throughput 0.54 0.60 0.571 High

847735 P63104 1433Z 2 High-throughput 0.54 0.60 0.571 High

864389 Q05209 PTN12 2 High-throughput 0.54 0.60 0.571 High

864571 Q99708 CTIP 2 Small-scale 0.53 0.60 0.563 High

864423 Q13901 C1D 1 Small-scale 0.57 0.55 0.561 High

232017 O60934 NBN 1 Small-scale 0.52 0.60 0.561 High

389437 P06401 PRGR 1 Small-scale 0.52 0.60 0.561 High

467597 P10827 THA 1 Small-scale 0.52 0.60 0.561 High

467667 P10828 THB 1 Small-scale 0.52 0.60 0.561 High

502623 P14635 CCNB1 1 Small-scale 0.52 0.60 0.561 High

519903 P16403 H12 1 Small-scale 0.52 0.60 0.561 High

864431 Q14676 MDC1 1 Small-scale 0.52 0.60 0.561 High

864434 Q14686 NCOA6 1 Small-scale 0.52 0.60 0.561 High

864464 Q5H9R7 PP6R3 1 Small-scale 0.52 0.60 0.561 High

864656 Q9UPN7 PP6R1 1 Small-scale 0.52 0.60 0.561 High

457427 P10398 ARAF 2 Small-scale 0.57 0.55 0.559 High

537628 P18887 XRCC1 2 Small-scale 0.57 0.55 0.559 High

563318 P22415 USF1 1 Small-scale 0.51 0.60 0.554 High

585492 P25490 TYY1 1 Small-scale 0.51 0.60 0.554 High

778070 P51858 HDGF 1 High-throughput 0.51 0.60 0.554 High

804691 P56180 TPTE 1 High-throughput 0.51 0.60 0.554 High

864458 Q3KP44 ANR55 1 High-throughput 0.38 0.80 0.553 High

864622 Q9HBH0 RHOF 1 High-throughput 0.38 0.80 0.553 High

505820 P14921 ETS1 1 Small-scale 0.50 0.60 0.550 High

530945 P17947 SPI1 1 Small-scale 0.50 0.60 0.550 High

768147 P50549 ETV1 1 Small-scale 0.50 0.60 0.550 High

864538 Q92934 BAD 2 High-throughput 0.54 0.55 0.547 High

864583 Q9BUH6 PAXX 2 Small-scale 0.54 0.55 0.547 High

864582 Q9BUB5 MKNK1 1 High-throughput 0.49 0.60 0.541 High

294772 O95365 ZBT7A 1 Small-scale 0.52 0.55 0.537 High

864393 Q08211 DHX9 1 Small-scale 0.52 0.55 0.537 High

159089 O15111 IKKA 1 Small-scale 0.48 0.60 0.536 High

145296 O14733 MP2K7 1 High-throughput 0.48 0.60 0.536 High

148630 O14829 PPE1 1 High-throughput 0.48 0.60 0.536 High

200624 O43741 AAKB2 1 High-throughput 0.48 0.60 0.536 High

205274 O43918 AIRE 1 Small-scale 0.48 0.60 0.536 High

255078 O75582 KS6A5 1 High-throughput 0.48 0.60 0.536 High

338788 P02545 LMNA 1 Small-scale 0.48 0.60 0.536 High

408691 P08069 IGF1R 1 High-throughput 0.48 0.60 0.536 High

411039 P08238 HS90B 1 Small-scale 0.48 0.60 0.536 High

512509 P15735 PHKG2 1 High-throughput 0.48 0.60 0.536 High

565119 P22612 KAPCG 1 High-throughput 0.48 0.60 0.536 High

611953 P29323 EPHB2 1 High-throughput 0.48 0.60 0.536 High

623786 P31152 MK04 1 High-throughput 0.48 0.60 0.536 High

668000 P35916 VGFR3 1 High-throughput 0.48 0.60 0.536 High

725652 P43250 GRK6 1 High-throughput 0.48 0.60 0.536 High

757039 P49450 CENPA 1 Small-scale 0.48 0.60 0.536 High

778609 P51957 NEK4 1 High-throughput 0.48 0.60 0.536 High

863333 P78368 KC1G2 1 High-throughput 0.48 0.60 0.536 High

864384 Q02763 TIE2 1 High-throughput 0.48 0.60 0.536 High

864420 Q13573 SNW1 1 High-throughput 0.48 0.60 0.536 High

864428 Q14457 BECN1 1 High-throughput 0.48 0.60 0.536 High

864446 Q15208 STK38 1 High-throughput 0.48 0.60 0.536 High

864454 Q16832 DDR2 1 High-throughput 0.48 0.60 0.536 High

864473 Q6J9G0 STYK1 1 High-throughput 0.48 0.60 0.536 High

864476 Q6NXR4 TTI2 1 Small-scale 0.48 0.60 0.536 High

864488 Q7Z695 ADCK2 1 High-throughput 0.48 0.60 0.536 High

864490 Q86U44 MTA70 1 High-throughput 0.48 0.60 0.536 High

864492 Q86UX6 ST32C 1 High-throughput 0.48 0.60 0.536 High

864516 Q8NI60 COQ8A 1 High-throughput 0.48 0.60 0.536 High

864537 Q92905 CSN5 1 High-throughput 0.48 0.60 0.536 High

864540 Q93034 CUL5 1 High-throughput 0.48 0.60 0.536 High

864547 Q96DB2 HDA11 1 High-throughput 0.48 0.60 0.536 High

864558 Q96QS6 KPSH2 1 High-throughput 0.48 0.60 0.536 High

864561 Q96RR4 KKCC2 1 High-throughput 0.48 0.60 0.536 High

864567 Q99615 DNJC7 1 High-throughput 0.48 0.60 0.536 High

864568 Q99640 PMYT1 1 High-throughput 0.48 0.60 0.536 High

864592 Q9BZR8 B2L14 1 High-throughput 0.48 0.60 0.536 High

864604 Q9H1C4 UN93B 1 High-throughput 0.48 0.60 0.536 High

864614 Q9H816 DCR1B 1 High-throughput 0.48 0.60 0.536 High

864624 Q9HCE5 MET14 1 High-throughput 0.48 0.60 0.536 High

864627 Q9NQB0 TF7L2 1 High-throughput 0.48 0.60 0.536 High

864633 Q9NRM7 LATS2 1 High-throughput 0.48 0.60 0.536 High

864637 Q9NSY0 NRBP2 1 High-throughput 0.48 0.60 0.536 High

864662 Q9Y265 RUVB1 1 Small-scale 0.48 0.60 0.536 High

864663 Q9Y275 TN13B 1 High-throughput 0.48 0.60 0.536 High

864677 Q9Y6S9 RPKL1 1 High-throughput 0.48 0.60 0.536 High

266687 O76064 RNF8 1 High-throughput 0.44 0.60 0.515 High

711607 P41002 CCNF 1 High-throughput 0.44 0.60 0.515 High

864495 Q8IVF5 TIAM2 1 High-throughput 0.44 0.60 0.515 High

864527 Q8WXH0 SYNE2 1 High-throughput 0.44 0.60 0.515 High

864676 Q9Y6N6 LAMC3 1 High-throughput 0.44 0.60 0.515 High

775639 P51617 IRAK1 1 High-throughput 0.48 0.55 0.513 High

864440 Q15051 IQCB1 1 High-throughput 0.48 0.55 0.513 High

864449 Q15746 MYLK 1 High-throughput 0.48 0.55 0.513 High

864580 Q9BSB4 ATGA1 1 High-throughput 0.48 0.55 0.513 High

864581 Q9BSI4 TINF2 1 High-throughput 0.48 0.55 0.513 High

864585 Q9BW61 DDA1 1 High-throughput 0.48 0.55 0.513 High

864611 Q9H5K3 SG196 1 High-throughput 0.48 0.55 0.513 High

864639 Q9NUX5 POTE1 1 High-throughput 0.48 0.55 0.513 High

864673 Q9Y679 AUP1 1 High-throughput 0.48 0.55 0.513 High

150421 O14920 IKKB 1 Small-scale 0.42 0.60 0.501 High

168341 O15379 HDAC3 1 Small-scale 0.42 0.60 0.501 High

355493 P04150 GCR 1 Small-scale 0.42 0.60 0.501 High

425371 P09651 ROA1 1 Small-scale 0.42 0.60 0.501 High

454954 P10144 GRAB 1 Small-scale 0.42 0.60 0.501 High

501828 P14598 NCF1 1 Small-scale 0.42 0.60 0.501 High

545215 P19878 NCF2 1 Small-scale 0.42 0.60 0.501 High

731687 P45983 MK08 1 Small-scale 0.42 0.60 0.501 High

761656 P49759 CLK1 1 High-throughput 0.42 0.60 0.501 High

763223 P49840 GSK3A 1 Small-scale 0.42 0.60 0.501 High

763551 P49841 GSK3B 1 Small-scale 0.42 0.60 0.501 High

784681 P52945 PDX1 1 Small-scale 0.42 0.60 0.501 High

796257 P54619 AAKG1 1 Small-scale 0.42 0.60 0.501 High

814846 P60484 PTEN 1 High-throughput 0.42 0.60 0.501 High

864377 Q00613 HSF1 1 Small-scale 0.42 0.60 0.501 High

864383 Q02539 H11 1 Small-scale 0.42 0.60 0.501 High

864419 Q13541 4EBP1 1 Small-scale 0.42 0.60 0.501 High

864444 Q15080 NCF4 1 Small-scale 0.42 0.60 0.501 High

864526 Q8WXE1 ATRIP 1 Small-scale 0.42 0.60 0.501 High

864535 Q92830 KAT2A 1 Small-scale 0.42 0.60 0.501 High

864667 Q9Y4B6 DCAF1 1 Small-scale 0.42 0.60 0.501 High

864671 Q9Y5B0 CTDP1 1 Small-scale 0.42 0.60 0.501 High

424555 P09619 PGFRB 1 High-throughput 0.42 0.60 0.500 High

456716 P10276 RARA 1 Small-scale 0.42 0.60 0.500 High

557802 P21802 FGFR2 1 High-throughput 0.42 0.60 0.500 High

864391 Q06418 TYRO3 1 High-throughput 0.42 0.60 0.500 High

864450 Q16288 NTRK3 1 High-throughput 0.42 0.60 0.500 High

864645 Q9UBX2 DUX4 1 High-throughput 0.44 0.55 0.494 High

157700 O15056 SYNJ2 1 High-throughput 0.44 0.55 0.493 High

401111 P07355 ANXA2 1 High-throughput 0.44 0.55 0.493 High

600137 P27487 DPP4 1 High-throughput 0.44 0.55 0.493 High

864474 Q6NUJ5 PWP2B 1 High-throughput 0.44 0.55 0.493 High

864591 Q9BZE4 GTPB4 1 High-throughput 0.44 0.55 0.493 High

864443 Q15077 P2RY6 1 High-throughput 0.48 0.50 0.489 High

864569 Q99677 LPAR4 1 High-throughput 0.48 0.50 0.489 High

340975 P02686 MBP 1 Small-scale 0.42 0.55 0.480 High

405898 P07910 HNRPC 1 Small-scale 0.42 0.55 0.480 High

407607 P07954 FUMH 1 Small-scale 0.42 0.55 0.480 High

408145 P08047 SP1 1 Small-scale 0.42 0.55 0.480 High

850749 P63279 UBC9 1 Small-scale 0.42 0.55 0.480 High

193139 O43439 MTG8R 1 Small-scale 0.42 0.55 0.479 High

118945 O00308 WWP2 1 High-throughput 0.38 0.60 0.479 High

141189 O14578 CTRO 1 High-throughput 0.38 0.60 0.479 High

157325 O15047 SET1A 1 High-throughput 0.38 0.60 0.479 High

163284 O15218 GP182 1 High-throughput 0.38 0.60 0.479 High

168931 O15393 TMPS2 1 High-throughput 0.38 0.60 0.479 High

188377 O43193 MTLR 1 High-throughput 0.38 0.60 0.479 High

198254 O43663 PRC1 1 High-throughput 0.38 0.60 0.479 High

211796 O60216 RAD21 1 High-throughput 0.38 0.60 0.479 High

220024 O60346 PHLP1 1 High-throughput 0.38 0.60 0.479 High

241144 O75147 OBSL1 1 High-throughput 0.38 0.60 0.479 High

251806 O75494 SRS10 1 Small-scale 0.38 0.60 0.479 High

278351 O94761 RECQ4 1 High-throughput 0.38 0.60 0.479 High

279174 O94762 RECQ5 1 Small-scale 0.38 0.60 0.479 High

284961 O94972 TRI37 1 High-throughput 0.38 0.60 0.479 High

291490 O95235 KI20A 1 High-throughput 0.38 0.60 0.479 High

304596 O95793 STAU1 1 High-throughput 0.38 0.60 0.479 High

306381 O95817 BAG3 1 High-throughput 0.38 0.60 0.479 High

308574 O95863 SNAI1 1 High-throughput 0.38 0.60 0.479 High

331523 P01137 TGFB1 1 High-throughput 0.38 0.60 0.479 High

357481 P04198 MYCN 1 High-throughput 0.38 0.60 0.479 High

358308 P04201 MAS 1 High-throughput 0.38 0.60 0.479 High

365891 P04792 HSPB1 1 Small-scale 0.38 0.60 0.479 High

368840 P04908 H2A1B 1 High-throughput 0.38 0.60 0.479 High

370282 P05023 AT1A1 1 High-throughput 0.38 0.60 0.479 High

402537 P07550 ADRB2 1 High-throughput 0.38 0.60 0.479 High

403355 P07711 CATL1 1 High-throughput 0.38 0.60 0.479 High

405052 P07900 HS90A 1 Small-scale 0.38 0.60 0.479 High

418391 P08922 ROS1 1 High-throughput 0.38 0.60 0.479 High

423066 P09467 F16P1 1 High-throughput 0.38 0.60 0.479 High

483477 P12004 PCNA 1 Small-scale 0.38 0.60 0.479 High

497194 P13693 TCTP 1 High-throughput 0.38 0.60 0.479 High

510926 P15374 UCHL3 1 Small-scale 0.38 0.60 0.479 High

531007 P17948 VGFR1 1 High-throughput 0.38 0.60 0.479 High

542064 P19438 TNR1A 1 High-throughput 0.38 0.60 0.479 High

548027 P20290 BTF3 1 High-throughput 0.38 0.60 0.479 High

548396 P20309 ACM3 1 High-throughput 0.38 0.60 0.479 High

548489 P20333 TNR1B 1 High-throughput 0.38 0.60 0.479 High

555393 P21462 FPR1 1 High-throughput 0.38 0.60 0.479 High

558479 P21917 DRD4 1 High-throughput 0.38 0.60 0.479 High

564713 P22607 FGFR3 1 High-throughput 0.38 0.60 0.479 High

565964 P22694 KAPCB 1 Small-scale 0.38 0.60 0.479 High

568295 P23246 SFPQ 1 Small-scale 0.38 0.60 0.479 High

579333 P24941 CDK2 1 High-throughput 0.38 0.60 0.479 High

596370 P26718 NKG2D 1 High-throughput 0.38 0.60 0.479 High

603247 P27816 MAP4 1 High-throughput 0.38 0.60 0.479 High

607311 P28340 DPOD1 1 High-throughput 0.38 0.60 0.479 High

864461 Q504Q3 PAN2 1 High-throughput 0.38 0.60 0.479 High

864658 Q9UPT9 UBP22 1 Small-scale 0.38 0.60 0.479 High

864659 Q9UQE7 SMC3 1 High-throughput 0.38 0.60 0.479 High

864660 Q9UQL6 HDAC5 1 High-throughput 0.38 0.60 0.479 High

864664 Q9Y2I6 NINL 1 High-throughput 0.38 0.60 0.479 High

612670 P29371 NK3R 1 High-throughput 0.38 0.60 0.479 High

620136 P30530 UFO 1 High-throughput 0.38 0.60 0.479 High

628129 P31946 1433B 1 High-throughput 0.38 0.60 0.479 High

629399 P31948 STIP1 1 High-throughput 0.38 0.60 0.479 High

649635 P33778 H2B1B 1 High-throughput 0.38 0.60 0.479 High

655023 P34925 RYK 1 Small-scale 0.38 0.60 0.479 High

655723 P34947 GRK5 1 High-throughput 0.38 0.60 0.479 High

657993 P35222 CTNB1 1 High-throughput 0.38 0.60 0.479 High

658668 P35226 BMI1 1 Small-scale 0.38 0.60 0.479 High

659468 P35228 NOS2 1 High-throughput 0.38 0.60 0.479 High

660836 P35244 RFA3 1 High-throughput 0.38 0.60 0.479 High

668375 P35968 VGFR2 1 High-throughput 0.38 0.60 0.479 High

675644 P36888 FLT3 1 High-throughput 0.38 0.60 0.479 High

675780 P36894 BMR1A 1 High-throughput 0.38 0.60 0.479 High

678368 P37275 ZEB1 1 Small-scale 0.38 0.60 0.479 High

708903 P40692 MLH1 1 High-throughput 0.38 0.60 0.479 High

727197 P43361 MAGA8 1 High-throughput 0.38 0.60 0.479 High

729905 P43694 GATA4 1 High-throughput 0.38 0.60 0.479 High

730133 P43699 NKX21 1 High-throughput 0.38 0.60 0.479 High

732514 P46059 S15A1 1 High-throughput 0.38 0.60 0.479 High

733197 P46100 ATRX 1 Small-scale 0.38 0.60 0.479 High

738126 P46821 MAP1B 1 High-throughput 0.38 0.60 0.479 High

738893 P46937 YAP1 1 High-throughput 0.38 0.60 0.479 High

752953 P49116 NR2C2 1 High-throughput 0.38 0.60 0.479 High

755218 P49336 CDK8 1 Small-scale 0.38 0.60 0.479 High

760388 P49736 MCM2 1 High-throughput 0.38 0.60 0.479 High

782393 P52701 MSH6 1 Small-scale 0.38 0.60 0.479 High

794922 P54274 TERF1 1 Small-scale 0.38 0.60 0.479 High

795175 P54278 PMS2 1 High-throughput 0.38 0.60 0.479 High

796012 P54578 UBP14 1 High-throughput 0.38 0.60 0.479 High

827945 P61981 1433G 1 High-throughput 0.38 0.60 0.479 High

851402 P67775 PP2AA 1 Small-scale 0.38 0.60 0.479 High

854544 P68400 CSK21 1 Small-scale 0.38 0.60 0.479 High

864374 P84243 H33 1 Small-scale 0.38 0.60 0.479 High

864376 Q00341 VIGLN 1 Small-scale 0.38 0.60 0.479 High

864379 Q01201 RELB 1 High-throughput 0.38 0.60 0.479 High

864381 Q01860 PO5F1 1 High-throughput 0.38 0.60 0.479 High

864382 Q02241 KIF23 1 High-throughput 0.38 0.60 0.479 High

864385 Q03060 CREM 1 Small-scale 0.38 0.60 0.479 High

864388 Q04912 RON 1 High-throughput 0.38 0.60 0.479 High

864392 Q07817 B2CL1 1 High-throughput 0.38 0.60 0.479 High

864394 Q08945 SSRP1 1 High-throughput 0.38 0.60 0.479 High

864399 Q12800 TFCP2 1 High-throughput 0.38 0.60 0.479 High

864400 Q12866 MERTK 1 High-throughput 0.38 0.60 0.479 High

864401 Q12873 CHD3 1 High-throughput 0.38 0.60 0.479 High

864404 Q12948 FOXC1 1 High-throughput 0.38 0.60 0.479 High

864405 Q12986 NFX1 1 High-throughput 0.38 0.60 0.479 High

864406 Q13111 CAF1A 1 Small-scale 0.38 0.60 0.479 High

864416 Q13451 FKBP5 1 High-throughput 0.38 0.60 0.479 High

864417 Q13469 NFAC2 1 High-throughput 0.38 0.60 0.479 High

864418 Q13489 BIRC3 1 High-throughput 0.38 0.60 0.479 High

864421 Q13616 CUL1 1 Small-scale 0.38 0.60 0.479 High

864424 Q14134 TRI29 1 Small-scale 0.38 0.60 0.479 High

864426 Q14258 TRI25 1 High-throughput 0.38 0.60 0.479 High

864427 Q14318 FKBP8 1 High-throughput 0.38 0.60 0.479 High

864429 Q14498 RBM39 1 High-throughput 0.38 0.60 0.479 High

864432 Q14683 SMC1A 1 High-throughput 0.38 0.60 0.479 High

864435 Q14839 CHD4 1 High-throughput 0.38 0.60 0.479 High

864439 Q15029 U5S1 1 High-throughput 0.38 0.60 0.479 High

864441 Q15052 ARHG6 1 Small-scale 0.38 0.60 0.479 High

864442 Q15058 KIF14 1 High-throughput 0.38 0.60 0.479 High

864445 Q15185 TEBP 1 High-throughput 0.38 0.60 0.479 High

864447 Q15365 PCBP1 1 Small-scale 0.38 0.60 0.479 High

864451 Q16526 CRY1 1 High-throughput 0.38 0.60 0.479 High

864453 Q16778 H2B2E 1 High-throughput 0.38 0.60 0.479 High

864459 Q49AN0 CRY2 1 High-throughput 0.38 0.60 0.479 High

864460 Q4G0J3 LARP7 1 High-throughput 0.38 0.60 0.479 High

864468 Q5U5Q3 MEX3C 1 Small-scale 0.38 0.60 0.479 High

864469 Q5XPI4 RN123 1 High-throughput 0.38 0.60 0.479 High

864477 Q6P1J9 CDC73 1 High-throughput 0.38 0.60 0.479 High

864481 Q6ZMQ8 LMTK1 1 High-throughput 0.38 0.60 0.479 High

864485 Q76L83 ASXL2 1 High-throughput 0.38 0.60 0.479 High

864487 Q7Z3E1 PARPT 1 High-throughput 0.38 0.60 0.479 High

864491 Q86UK7 ZN598 1 High-throughput 0.38 0.60 0.479 High

864498 Q8IWR0 Z3H7A 1 High-throughput 0.38 0.60 0.479 High

864499 Q8IWU2 LMTK2 1 High-throughput 0.38 0.60 0.479 High

864500 Q8IXJ9 ASXL1 1 High-throughput 0.38 0.60 0.479 High

864501 Q8IY34 S15A3 1 High-throughput 0.38 0.60 0.479 High

864502 Q8IZD6 S22AF 1 High-throughput 0.38 0.60 0.479 High

864503 Q8N386 LRC25 1 High-throughput 0.38 0.60 0.479 High

864504 Q8N3U4 STAG2 1 High-throughput 0.38 0.60 0.479 High

864507 Q8N5Y2 MS3L1 1 Small-scale 0.38 0.60 0.479 High

864517 Q8TAD8 SNIP1 1 High-throughput 0.38 0.60 0.479 High

864522 Q8TEK3 DOT1L 1 High-throughput 0.38 0.60 0.479 High

864523 Q8WVJ2 NUDC2 1 High-throughput 0.38 0.60 0.479 High

864530 Q92560 BAP1 1 High-throughput 0.38 0.60 0.479 High

864531 Q92630 DYRK2 1 High-throughput 0.38 0.60 0.479 High

864534 Q92769 HDAC2 1 High-throughput 0.38 0.60 0.479 High

864536 Q92858 ATOH1 1 Small-scale 0.38 0.60 0.479 High

864539 Q92993 KAT5 1 Small-scale 0.38 0.60 0.479 High

864542 Q96AQ6 PBIP1 1 High-throughput 0.38 0.60 0.479 High

864544 Q96CF2 CHM4C 1 High-throughput 0.38 0.60 0.479 High

864548 Q96DN6 MBD6 1 High-throughput 0.38 0.60 0.479 High

864550 Q96EA4 SPDLY 1 High-throughput 0.38 0.60 0.479 High

864551 Q96FA3 PELI1 1 Small-scale 0.38 0.60 0.479 High

864554 Q96HY6 DDRGK 1 High-throughput 0.38 0.60 0.479 High

864557 Q96PU4 UHRF2 1 Small-scale 0.38 0.60 0.479 High

864560 Q96RK0 CIC 1 High-throughput 0.38 0.60 0.479 High

864563 Q96SN8 CK5P2 1 Small-scale 0.38 0.60 0.479 High

864564 Q96T51 RUFY1 1 High-throughput 0.38 0.60 0.479 High

864572 Q99759 M3K3 1 High-throughput 0.38 0.60 0.479 High

864573 Q99814 EPAS1 1 Small-scale 0.38 0.60 0.479 High

864575 Q99873 ANM1 1 High-throughput 0.38 0.60 0.479 High

864576 Q99986 VRK1 1 High-throughput 0.38 0.60 0.479 High

864579 Q9BRS2 RIOK1 1 High-throughput 0.38 0.60 0.479 High

864586 Q9BWK5 CYREN 1 Small-scale 0.38 0.60 0.479 High

864589 Q9BXW9 FACD2 1 High-throughput 0.38 0.60 0.479 High

864600 Q9H0M0 WWP1 1 Small-scale 0.38 0.60 0.479 High

864603 Q9H0X4 F234A 1 High-throughput 0.38 0.60 0.479 High

864606 Q9H444 CHM4B 1 High-throughput 0.38 0.60 0.479 High

864609 Q9H4L7 SMRCD 1 High-throughput 0.38 0.60 0.479 High

864613 Q9H7Z6 KAT8 1 Small-scale 0.38 0.60 0.479 High

864615 Q9H871 RMD5A 1 High-throughput 0.38 0.60 0.479 High

864617 Q9H8V3 ECT2 1 High-throughput 0.38 0.60 0.479 High

864626 Q9NPI1 BRD7 1 High-throughput 0.38 0.60 0.479 High

864631 Q9NRC8 SIR7 1 High-throughput 0.38 0.60 0.479 High

864634 Q9NRZ9 HELLS 1 Small-scale 0.38 0.60 0.479 High

864641 Q9NWS0 PIHD1 1 High-throughput 0.38 0.60 0.479 High

864643 Q9NZQ7 PD1L1 1 High-throughput 0.38 0.60 0.479 High

864644 Q9UBN7 HDAC6 1 Small-scale 0.38 0.60 0.479 High

864646 Q9UGI0 ZRAN1 1 High-throughput 0.38 0.60 0.479 High

864648 Q9UHD9 UBQL2 1 High-throughput 0.38 0.60 0.479 High

864649 Q9UHV7 MED13 1 Small-scale 0.38 0.60 0.479 High

864650 Q9UJX6 ANC2 1 Small-scale 0.38 0.60 0.479 High

864651 Q9UK00 CC018 1 High-throughput 0.38 0.60 0.479 High

864652 Q9ULB1 NRX1A 1 High-throughput 0.38 0.60 0.479 High

864653 Q9ULY5 CLC4E 1 High-throughput 0.38 0.60 0.479 High

864655 Q9UMW8 UBP18 1 Small-scale 0.38 0.60 0.479 High

864657 Q9UPN9 TRI33 1 High-throughput 0.38 0.60 0.479 High

864670 Q9Y572 RIPK3 1 High-throughput 0.38 0.60 0.479 High

864674 Q9Y6B6 SAR1B 1 High-throughput 0.38 0.60 0.479 High

311567 O95994 AGR2 1 High-throughput 0.44 0.50 0.470 High

404550 P07858 CATB 1 High-throughput 0.44 0.50 0.470 High

589434 P25774 CATS 1 High-throughput 0.44 0.50 0.470 High

754679 P49286 MTR1B 1 High-throughput 0.43 0.50 0.464 High

59976 A6NDX5 ZN840 1 High-throughput 0.38 0.55 0.458 High

65194 A8MVS5 HIDE1 1 High-throughput 0.38 0.55 0.458 High

142111 O14579 COPE 1 High-throughput 0.38 0.55 0.458 High

173507 O15541 R113A 1 High-throughput 0.38 0.55 0.458 High

220548 O60356 NUPR1 1 High-throughput 0.38 0.55 0.458 High

228006 O60829 PAGE4 1 High-throughput 0.38 0.55 0.458 High

281850 O94874 UFL1 1 High-throughput 0.38 0.55 0.458 High

285563 O94985 CSTN1 1 High-throughput 0.38 0.55 0.458 High

286300 O94992 HEXI1 1 High-throughput 0.38 0.55 0.458 High

288801 O95163 ELP1 1 Small-scale 0.38 0.55 0.458 High

296885 O95433 AHSA1 1 Small-scale 0.38 0.55 0.458 High

310742 O95985 TOP3B 1 High-throughput 0.38 0.55 0.458 High

341954 P02751 FINC 1 High-throughput 0.38 0.55 0.458 High

428749 P09958 FURIN 1 High-throughput 0.38 0.55 0.458 High

487071 P12755 SKI 1 Small-scale 0.38 0.55 0.458 High

495553 P13612 ITA4 1 High-throughput 0.38 0.55 0.458 High

515382 P16050 LOX15 1 Small-scale 0.38 0.55 0.458 High

520570 P16455 MGMT 1 Small-scale 0.38 0.55 0.458 High

524660 P17096 HMGA1 1 High-throughput 0.38 0.55 0.458 High

659721 P35232 PHB1 1 High-throughput 0.38 0.55 0.458 High

717906 P42167 LAP2B 1 High-throughput 0.38 0.55 0.458 High

756764 P49427 UB2R1 1 High-throughput 0.38 0.55 0.458 High

759659 P49711 CTCF 1 Small-scale 0.38 0.55 0.458 High

766532 P50402 EMD 1 High-throughput 0.38 0.55 0.458 High

784967 P53007 TXTP 1 High-throughput 0.38 0.55 0.458 High

793518 P54252 ATX3 1 High-throughput 0.38 0.55 0.458 High

809210 P57740 NU107 1 High-throughput 0.38 0.55 0.458 High

814354 P60468 SC61B 1 High-throughput 0.38 0.55 0.458 High

842533 P62877 RBX1 1 High-throughput 0.38 0.55 0.458 High

864375 P98153 IDD 1 High-throughput 0.38 0.55 0.458 High

864380 Q01628 IFM3 1 High-throughput 0.38 0.55 0.458 High

864390 Q05923 DUS2 1 High-throughput 0.38 0.55 0.458 High

864398 Q10571 MN1 1 Small-scale 0.38 0.55 0.458 High

864407 Q13155 AIMP2 1 High-throughput 0.38 0.55 0.458 High

864436 Q14872 MTF1 1 High-throughput 0.38 0.55 0.458 High

864437 Q14999 CUL7 1 High-throughput 0.38 0.55 0.458 High

864448 Q15628 TRADD 1 High-throughput 0.38 0.55 0.458 High

864455 Q29RF7 PDS5A 1 High-throughput 0.38 0.55 0.458 High

864471 Q69YN4 VIR 1 High-throughput 0.38 0.55 0.458 High

864475 Q6NUN9 ZN746 1 High-throughput 0.38 0.55 0.458 High

864478 Q6P2Q9 PRP8 1 High-throughput 0.38 0.55 0.458 High

864484 Q75N03 HAKAI 1 Small-scale 0.38 0.55 0.458 High

864493 Q86UY6 NAA40 1 High-throughput 0.38 0.55 0.458 High

864506 Q8N4V1 EMC5 1 High-throughput 0.38 0.55 0.458 High

864509 Q8N766 EMC1 1 High-throughput 0.38 0.55 0.458 High

864515 Q8NFH5 NUP35 1 High-throughput 0.38 0.55 0.458 High

864521 Q8TEE9 SAP25 1 Small-scale 0.38 0.55 0.458 High

864529 Q8WYQ5 DGCR8 1 Small-scale 0.38 0.55 0.458 High

864543 Q96C86 DCPS 1 High-throughput 0.38 0.55 0.458 High

864546 Q96CW1 AP2M1 1 High-throughput 0.38 0.55 0.458 High

864552 Q96FF9 CDCA5 1 High-throughput 0.38 0.55 0.458 High

864565 Q96T60 PNKP 1 Small-scale 0.38 0.55 0.458 High

864570 Q99706 KI2L4 1 High-throughput 0.38 0.55 0.458 High

864584 Q9BW27 NUP85 1 High-throughput 0.38 0.55 0.458 High

864587 Q9BXB1 LGR4 1 High-throughput 0.38 0.55 0.458 High

864593 Q9BZV1 UBXN6 1 Small-scale 0.38 0.55 0.458 High

864595 Q9C0C9 UBE2O 1 High-throughput 0.38 0.55 0.458 High

864598 Q9H040 SPRTN 1 High-throughput 0.38 0.55 0.458 High

864599 Q9H0E2 TOLIP 1 High-throughput 0.38 0.55 0.458 High

864602 Q9H0W5 CCDC8 1 High-throughput 0.38 0.55 0.458 High

864605 Q9H1E3 NUCKS 1 High-throughput 0.38 0.55 0.458 High

864612 Q9H6Z9 EGLN3 1 High-throughput 0.38 0.55 0.458 High

864616 Q9H8T0 AKTIP 1 Small-scale 0.38 0.55 0.458 High

864625 Q9NP50 SHCAF 1 Small-scale 0.38 0.55 0.458 High

864628 Q9NQC7 CYLD 1 High-throughput 0.38 0.55 0.458 High

864629 Q9NQW6 ANLN 1 High-throughput 0.38 0.55 0.458 High

864632 Q9NRD1 FBX6 1 High-throughput 0.38 0.55 0.458 High

864636 Q9NS91 RAD18 1 High-throughput 0.38 0.55 0.458 High

864647 Q9UGI8 TES 1 High-throughput 0.38 0.55 0.458 High

864666 Q9Y3A5 SBDS 1 Small-scale 0.38 0.55 0.458 High

864668 Q9Y4K0 LOXL2 1 High-throughput 0.38 0.55 0.458 High

864672 Q9Y5B9 SP16H 1 High-throughput 0.38 0.55 0.458 High

864678 Q9Y6X3 SCC4 1 High-throughput 0.38 0.55 0.458 High

790791 P53804 TTC3 1 High-throughput 0.35 0.60 0.457 High

95505 F5H1C8 F5H1C8 1 High-throughput 0.33 0.60 0.445 High

289141 O95166 GBRAP 1 High-throughput 0.33 0.60 0.445 High

296252 O95409 ZIC2 1 Small-scale 0.33 0.60 0.445 High

442118 P0C0S8 H2A1 1 Small-scale 0.33 0.60 0.445 High

530898 P17936 IBP3 1 Small-scale 0.33 0.60 0.445 High

815307 P60520 GBRL2 1 High-throughput 0.33 0.60 0.445 High

864395 Q09161 NCBP1 1 High-throughput 0.33 0.60 0.445 High

864408 Q13185 CBX3 1 Small-scale 0.33 0.60 0.445 High

864410 Q13285 STF1 1 Small-scale 0.33 0.60 0.445 High

864433 Q14684 RRP1B 1 High-throughput 0.33 0.60 0.445 High

864533 Q92731 ESR2 1 High-throughput 0.33 0.60 0.445 High

864594 Q9C0B5 ZDHC5 1 High-throughput 0.33 0.60 0.445 High

864596 Q9GZQ8 MLP3B 1 High-throughput 0.33 0.60 0.445 High

864601 Q9H0R8 GBRL1 1 High-throughput 0.33 0.60 0.445 High

864607 Q9H492 MLP3A 1 High-throughput 0.33 0.60 0.445 High

864620 Q9H9Z2 LN28A 1 High-throughput 0.33 0.60 0.445 High

68341 B2Y833 B2Y833 1 Small-scale 0.39 0.50 0.442 High

120133 O00398 P2Y10 1 High-throughput 0.38 0.50 0.437 High

409954 P08173 ACM4 1 High-throughput 0.38 0.50 0.437 High

649074 P33681 CD80 1 High-throughput 0.38 0.50 0.437 High

753772 P49146 NPY2R 1 High-throughput 0.38 0.50 0.437 High

754066 P49190 PTH2R 1 High-throughput 0.38 0.50 0.437 High

783201 P52798 EFNA4 1 High-throughput 0.38 0.50 0.437 High

810136 P58400 NRX1B 1 High-throughput 0.38 0.50 0.437 High

864412 Q13304 GPR17 1 High-throughput 0.38 0.50 0.437 High

864430 Q14627 I13R2 1 High-throughput 0.38 0.50 0.437 High

864472 Q6EMK4 VASN 1 High-throughput 0.38 0.50 0.437 High

864479 Q6UX01 LMBRL 1 High-throughput 0.38 0.50 0.437 High

864480 Q6UY01 LRC31 1 High-throughput 0.38 0.50 0.437 High

864483 Q6ZTA4 TRI67 1 High-throughput 0.38 0.50 0.437 High

864486 Q7L273 KCTD9 1 High-throughput 0.38 0.50 0.437 High

864489 Q86T26 TM11B 1 High-throughput 0.38 0.50 0.437 High

864510 Q8N8L6 ARL10 1 High-throughput 0.38 0.50 0.437 High

864520 Q8TDB4 HUMMR 1 High-throughput 0.38 0.50 0.437 High

864528 Q8WXI8 CLC4D 1 High-throughput 0.38 0.50 0.437 High

864556 Q96PE5 OPALI 1 High-throughput 0.38 0.50 0.437 High

864610 Q9H4M7 PKHA4 1 High-throughput 0.38 0.50 0.437 High

864623 Q9HC73 CRLF2 1 High-throughput 0.38 0.50 0.437 High

864630 Q9NR31 SAR1A 1 High-throughput 0.38 0.50 0.437 High

864640 Q9NVX7 KBTB4 1 High-throughput 0.38 0.50 0.437 High

105861 K7EJ46 SIM22 1 Small-scale 0.33 0.55 0.426 High

371594 P05067 A4 1 Small-scale 0.33 0.55 0.426 High

546216 P20042 IF2B 1 Small-scale 0.33 0.55 0.426 High

602241 P27695 APEX1 1 Small-scale 0.33 0.55 0.426 High

812154 P60033 CD81 1 Small-scale 0.33 0.55 0.426 High

864462 Q53F19 NCBP3 1 High-throughput 0.33 0.55 0.426 High

864496 Q8IW19 APLF 1 Small-scale 0.33 0.55 0.426 High

864505 Q8N488 RYBP 1 High-throughput 0.33 0.55 0.426 High

864638 Q9NTX7 RN146 1 Small-scale 0.33 0.55 0.426 High

263229 O75943 RAD17 1 Small-scale 0.28 0.60 0.408 High

764825 P49959 MRE11 1 Small-scale 0.28 0.60 0.408 High

864397 Q10567 AP1B1 1 Small-scale 0.28 0.60 0.408 High

864470 Q66K89 E4F1 1 Small-scale 0.28 0.60 0.408 High

864635 Q9NS23 RASF1 1 Small-scale 0.28 0.60 0.408 High

769784 P50876 R144A 3 Small-scale 0.75 0.16 0.349 High

539473 P19320 VCAM1 3 High-throughput 0.67 0.16 0.330 High

864590 Q9BYF1 ACE2 2 High-throughput 0.57 0.16 0.305 High

244689 O75323 NIPS2 1 High-throughput 0.48 0.16 0.279 Low

864497 Q8IWF2 FXRD2 1 High-throughput 0.48 0.16 0.279 Low

864513 Q8NBM4 UBAC2 1 High-throughput 0.48 0.16 0.279 Low

864545 Q96CS3 FAF2 1 High-throughput 0.48 0.16 0.279 Low

864549 Q96DT6 ATG4C 1 High-throughput 0.48 0.16 0.279 Low

864555 Q96JQ5 M4A4A 1 High-throughput 0.48 0.16 0.279 Low

508720 P15169 CBPN 1 High-throughput 0.44 0.16 0.269 Low

864456 Q2M2D7 TBC28 1 High-throughput 0.44 0.16 0.269 Low

864457 Q3B820 F161A 1 High-throughput 0.44 0.16 0.269 Low

864466 Q5T5P2 SKT 1 High-throughput 0.44 0.16 0.269 Low

239488 O75078 ADA11 1 High-throughput 0.38 0.16 0.249 Low

505137 P14867 GBRA1 1 High-throughput 0.38 0.16 0.249 Low

864438 Q15006 EMC2 1 High-throughput 0.38 0.16 0.249 Low

864463 Q5BJD5 TM41B 1 High-throughput 0.38 0.16 0.249 Low

864511 Q8N9I9 DTX3 1 Small-scale 0.38 0.16 0.249 Low

864512 Q8NAX2 KDF1 1 High-throughput 0.38 0.16 0.249 Low

864518 Q8TB96 TIP 1 High-throughput 0.38 0.16 0.249 Low

864519 Q8TBB1 LNX1 1 Small-scale 0.38 0.16 0.249 Low

864524 Q8WVP5 TP8L1 1 Small-scale 0.38 0.16 0.249 Low

864532 Q92729 PTPRU 1 High-throughput 0.38 0.16 0.249 Low

864642 Q9NZD1 GPC5D 1 High-throughput 0.38 0.16 0.249 Low

864665 Q9Y312 AAR2 1 High-throughput 0.38 0.16 0.249 Low

217865 O60303 KATIP 1 High-throughput 0.33 0.16 0.232 Low

864467 Q5T7N2 LITD1 1 High-throughput 0.33 0.16 0.232 Low

864482 Q6ZNK6 TIFAB 1 High-throughput 0.33 0.16 0.232 Low

864514 Q8NCK7 MOT11 1 High-throughput 0.33 0.16 0.232 Low

*************Service provided by Combinatics************************
